# Doctors in Japanese prisoner-of-war camps in Taiwan in the Second World War and their personal accounts of captivity

**DOI:** 10.1017/mdh.2024.41

**Published:** 2025-04

**Authors:** Katherine M. Venables

**Affiliations:** St Cross College, University of Oxford, Oxford, UK

**Keywords:** Second World War, Taiwan, Japan, Allied prisoners-of-war, Doctors, Personal accounts

## Abstract

Taiwan became a Japanese colony in 1895 and in the Second World War was geographically central in Japan’s wartime possessions and strategically important, with military airfields, ports, and a copper mine. Its sixteen prisoner-of-war camps included four labour camps. Taiwan was also the first place to which senior officers and colonial officials were dispersed after the Allied surrenders in Hong Kong, Singapore, Indonesia and the Philippines. Forty-five doctors from the British, Australian, Dutch and American forces were identified who spent at least part of their captivity on Taiwan. This article uses their personal accounts, official documents and secondary sources to describe them and their work. Although the oldest had experience in the First World War and some had practised in the region, others were young, recently-qualified generalists. Most were transferred between several camps, with one consequence that few contemporaneous medical records survive. Doctors shared the risks and hardships of all prisoners: they lost weight and had the same nutritional disorders, infections and infestations as their patients. Two died. They became significant, scrutinised figures in the camps. Their patients valued their work and understood that they lacked resources for fully effective medical practice.

## Introduction

So, often still, I am shaken with wonder at being alive. … War taught me the fickle lightness of life, as prison camps taught me the frail tenacity of survival. Excellent to know one is worthless and alive.

David Piper. *I am Well, who are You?* ^1^

There are surprising gaps in the academic literature about prisoners of war (PoWs) of the Japanese in the Second World War despite many popular films and a plethora of life-writing. Primary sources are scarce: guards destroyed camp records at the end of the war, many Anglophone researchers find Japanese (and Dutch) documents difficult to access, and some British and American archives are not informatively catalogued; there is a ‘lack of documentary evidence and [a] breadth of anecdotal evidence.’^2^ There is also preferential selection. Commentators, such as Gill, note that imprisonment in Japan itself, or its possessions of Taiwan, Korea and Manchuria, is less well-documented than, for example, in the camps on the Thai-Burma railway.^3^ Flower argues that officers have received less attention than other ranks and are often disparaged or caricatured.^4^ Although Japanese war crimes could reasonably be summarised as ‘medical’, historians have studied the work of captured doctors only rarely: a history of US Army medicine during the war against the Japanese, for example, includes only twenty pages on PoWs,^5^ and Flower notes that an initial plan to add a volume on PoWs to the British official history was abandoned.^6^ There was a flurry of articles about disease and practice in the camps in medical journals immediately after the war, but the material was not pulled together until Gill’s 2009 thesis and 2017 book.^7^ Hearder published her book on captive Australian doctors in 2020.^8^

In recent decades, a focussed approach has provided insights. As well as Gill’s and Hearder’s work, Kovner, for example, emphasises the rivalries and bureaucratic obstacles at the top of the Japanese PoW administration,^9^ Michno documents the ‘hellship’ voyages,^10^ Tett has published a detailed six-volume postal history,^11^ Linton examines the work of the British War Crimes Courts in Hong Kong.^12^ This article takes a similar approach by examining a complete group of all the Allied military doctors who were imprisoned by the Japanese in Taiwan; it aims to describe the diversity of their experience, as well as its commonalities.

## Japan in the Second World War

The background to why these doctors were imprisoned in Taiwan stretches back to the nineteenth century when Japan became increasingly expansionist and competed commercially with European nations and America. Her military conflicts with imperial China, the old regional power, supplied her first overseas colony, Taiwan, in 1895 and Shirane’s wide-ranging history describes Taiwan’s role as the Japanese empire’s southern ‘gateway’.^13^ Japan then gained control of Korea (1910) and Manchuria (1931) and developed the concept of the ‘Greater East Asia Co-Prosperity Sphere’ in which, as leader, she would free East and South-East Asian nations from their colonial oppressors. By the outbreak of the Second World War in September 1939 Japan’s army was unbeaten in the wars with China and she had a well-equipped navy and air force. In 1940 she signed pacts with Germany and Italy, Vichy France and neutral Thailand.

Then, on 7 December 1941, Japan opened a stunning series of surprise attacks on strongholds around South-East Asia and the Pacific. The attack on Pearl Harbor in Hawaii brought America into the war. The Allies had under-estimated the Japanese military, often in racialised terms but, by May 1942, they had captured over 300,000 PoWs, mostly in four major surrenders: about 14,000 in Hong Kong in December 1941 (British, Canadian, Indian), 130,000 in Singapore in February 1942 (British, Australian, Indian), 32,000 in Java in March 1942 (Dutch, British, Australian, American), and 75,000 in the Philippines in April and May 1942 (American, Filipino).^14^

**Figure 1.**
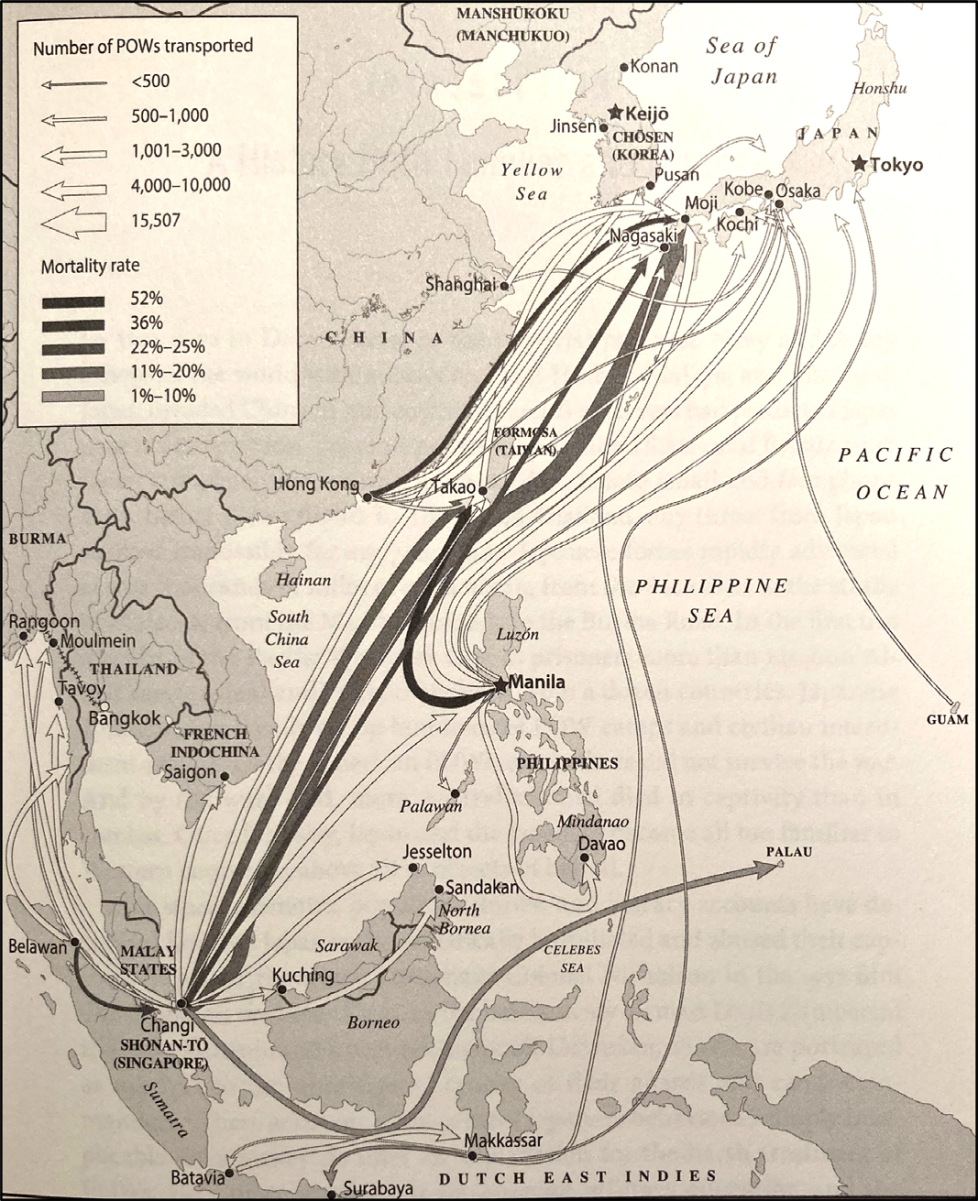
Map showing PoW movements around Japanese wartime possessions. From Kovner, *op. cit.* (note 9), with permission.

Hata summarises the evolution of Japanese attitudes to PoWs in the early twentieth century as ‘from consideration to contempt’.^15^ Kovner stresses that the small, centralised but bureaucratically confused Japanese PoW administration was not equipped to deal with so many captives, echoing Flower’s description of it as ‘secretive’.^16^ However, their labour would contribute to the war effort and propaganda using their images would demonstrate that Western imperialists could be defeated.^17^ From April 1942 onwards the Japanese dispersed their captives to what would become about 300 camps. Figure 1 shows the major sea transport routes and illustrates Taiwan’s central location in Japan’s wartime possessions.

## Taiwan

**Figure 2.**
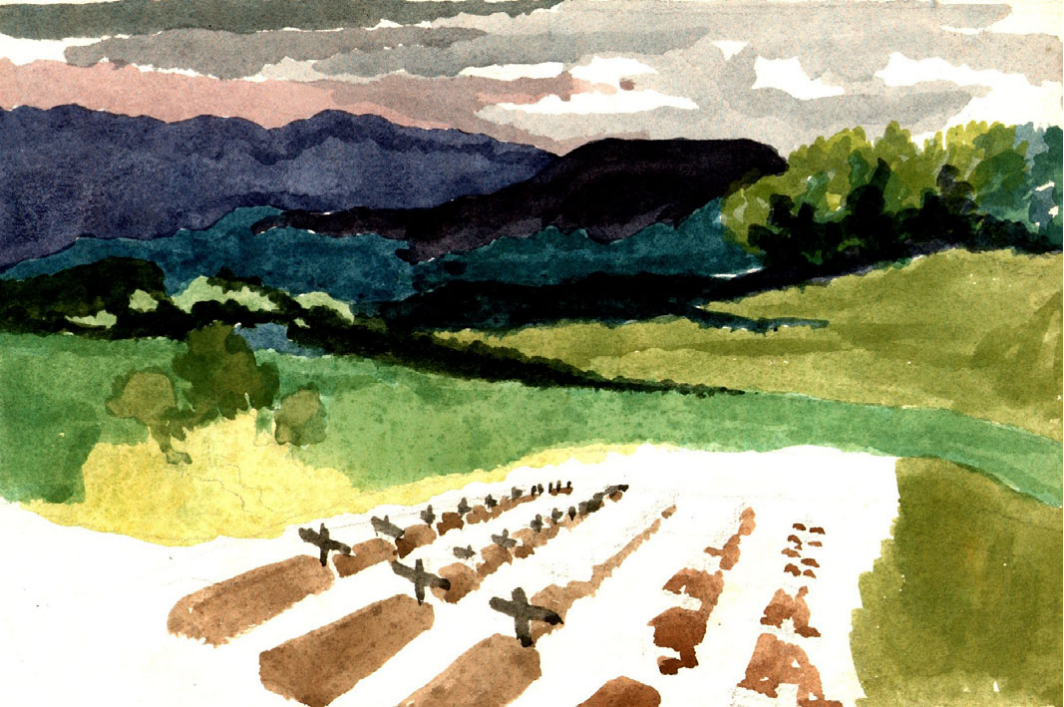
Cemetery and mountains at Shirakawa. Stallard, *op. cit.* (note 21), with permission.

Taiwan is approximately 245 by 90 miles and situated about 100 miles off the coast of southeastern China. Mountains cover two-thirds of the island’s surface, rising to over 10,000 feet. It has a subtropical climate with over 100 inches of rainfall annually, but weather conditions depend on altitude and the monsoon pattern. Its old Portuguese name, *Ilha Formosa*, meant ‘beautiful island’ and prisoners noticed the island’s ‘lush countryside’,^18^ its ‘hazy spray trees in the first sunlight’^19^ and mountains like ‘misty jade’,^20^ and sketched its landscapes (Figure 2).^21^

In August 1942, when the first party of PoWs arrived, only older Taiwanese could remember life before Japanese rule. Young Taiwanese men became camp guards, some to be tried later as war criminals.^22^ It had copper deposits, produced sugar, hosted military airfields, and its ports were stop-off points for naval and merchant ships. Thousands of PoWs were transported through the ports, confined in the holds of requisitioned cargo vessels, which became known as ‘hellships’ because of the conditions on board. Michno documents a total of 156 voyages around Japan’s wartime possessions in which c. 1,540 deaths are attributed to transport conditions alone.^23^ The ships also carried troops and war supplies but there were no indicators that they were used for prisoner transport and c. 19,000 died in Allied attacks. In part, this explains the often-quoted higher death rate of PoWs of the Japanese compared with those in the European theatre, a topic explored by Sturma.^24^

**Figure 3.**
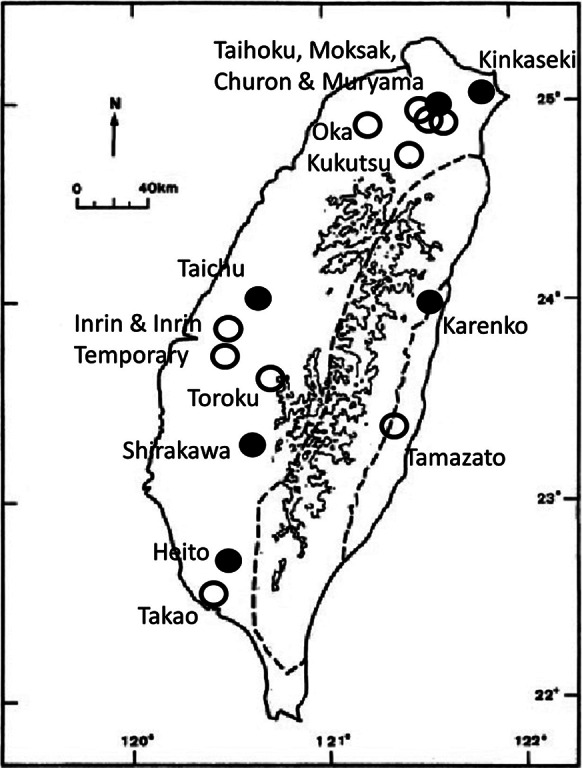
Map of Taiwan, showing the approximate location of PoW camps. Closed circle = major camp. Open circle = temporary camp. Contour lines at 2,000 and 3,000 metres. Dotted line shows boundary of Taiwan’s Backbone Range. Modified from Figure 2 in Yui and Chu, *op. cit.* (note 30), with permission.

The authorities in Tokyo wanted to place 7,000 PoWs in Taiwan to work in mining, construction and agriculture.^25^ A low estimate of the number actually incarcerated is 2,880, but others are higher, over 4,000.^26^ The camps were poorly documented until Hurst’s extensive oral history and archival research led to a memorial society, a website and a book.^27^ Hurst’s book is detailed, informative and comprehensively illustrated but does not use footnotes or references. Tett provides a summary description of the camps.^28^ Sixteen are listed in Table 1 in order of the date they opened.^29^ Their location is indicated in Figure 3.^30^ Taihoku and Taichu were built as PoW camps, but others were in repurposed buildings, for example, a military barracks (Karenko), a school (Toroku) and a hotel (Inrin). Kukutsu and Oka were constructed by PoWs. They can be grouped into labour camps, senior officers’ camps, and temporary holding camps.

**Table 1.** PoW camps in Taiwan

Camp	Comments	Date open	Date closed
**Heito**	PoWs cleared rocks for sugar cane and worked in a sugar factory. Closed after Allied bombing.	12 Aug 1942	15 Mar 1945
**Karenko**	First senior officers’ camp.	17 Aug 1942	25 Jun 1943
**Takao**	Transit camp near Takao harbour.	07 Sep 1942	15 Feb 1945
**Taichu**	PoWs excavated a flood diversion channel. Camp evacuated after flood.	27 Sep 1942	01 Jul 1944
**Taihoku**	PoWs worked on constructing a ‘Victory Park’ and in railway and bus repair shops.	14 Nov 1942	06 Sep 1945
**Kinkaseki**	Copper mining camp. PoWs moved to Kukutsu when it closed.	14 Nov 1942	16 May 1945
**Tamazato**	Temporary senior officers’ camp before Red Cross visit.	02 Apr 1943	24 Jun 1943
**Shirakawa**	Second senior officers’ camp. Hospital camp from 1944.	06 Jun 1943	26 Aug 1945
**Moksak**	Camp for the most senior officers.	24 Jun 1943	06 Dec 1944
**Inrin**	Camp for Taichu PoWs after flood.	01 Jul 1944	05 Mar 1945
**Inrin Temporary**	Subcamp of Inrin. Temporary camp for survivors of *Hokusen Maru.*	09 Nov 1944	15 Jan 1945
**Toroku**	Temporary camp for survivors of *Hokusen Maru.* Later housed other PoWs.	09 Nov 1944	11 Apr 1945
**Kukutsu**	Subcamp of Kinkaseki at the end of the war. Potential extermination camp.	16 May 1945	24 Aug 1945
**Oka**	Subcamp of Taihoku at the end of the war. Potential extermination camp.	12 Jun 1945	21 Aug 1945
**Churon**	Temporary pre-evacuation camp for PoWs from Kinkaseki after the Japanese surrender.	25 Aug 1945	06 Sep 1945
**Maruyama**	Temporary pre-evacuation camp for PoWs from Shirakawa after the Japanese surrender.	27 Aug 1945	06 Sep 1945

Modified from West Point Academy, *op. cit.* (note 29), public domain.

## The Taiwan doctors

The author searched the website of the *Taiwan POW Camps Memorial Society* for doctors using relevant search terms and compared the names with the 1944 list of Taiwan PoWs held in The National Archives which identified doctors by a handwritten Japanese character.^31^ Searches were carried out for newspaper items, obituaries, directory entries,^32^ and first-person material in diaries, memoirs, articles, theses, reports, and War Crimes Trial testimony. The indices of secondary histories were also searched. Forty-five doctors were imprisoned in Taiwan at some point but the number on the island at any one time was always lower than forty-five and probably lower than twenty. All are listed in Appendix 1. They arrived in Taiwan by sea.

Table 2 shows the number of doctors by country, branch of the military and rank. Four countries are represented, the largest group American (22), followed by 14 in the British forces (including a Canadian in the Indian Medical Service (IMS)), five from the Royal Netherlands East Indies Army and four from the Australian Imperial Force (AIF). Most (42/45) were army doctors. A surprisingly high proportion, 17/45, were full colonels (or their naval equivalent) and above. This unusual distribution is explained because from 1942 to 1944 the Japanese used Taiwan to imprison the most senior military officers and civilian colonial officials, an issue discussed in more detail below.

**Table 2.** Branch of service and rank of the Taiwan doctors

Branch of service	Rank							
	Maj-Gen.	Brigadier	Colonel*	Lt-Col.*	Major	Captain	Lieutenant	n
**Australian Army Medical Corps**			**3**			**1**		**4**
**Royal Netherlands Indies Army Medical Service**	**1**		**3**				**1**	**5**
**Total United Kingdom medical:**		**1**	**4**	**2**	**2**	**5**		**14**
Royal Army Medical Corps		1	3			5		9
Indian Medical Service†			1	1	2†			4
Royal Naval Medical Service				1				1
**Total United States medical:**			**5**	**2**	**2**	**11**	**2**	**22**
United States Army Medical Corps			3	2	2	11	2	20
United States Navy Medical Corps			2					2
**Total**	**1**	**1**	**15**	**4**	**4**	**17**	**3**	**45**

* or the equivalent naval rank; † includes a Canadian.

**Figure 4.**
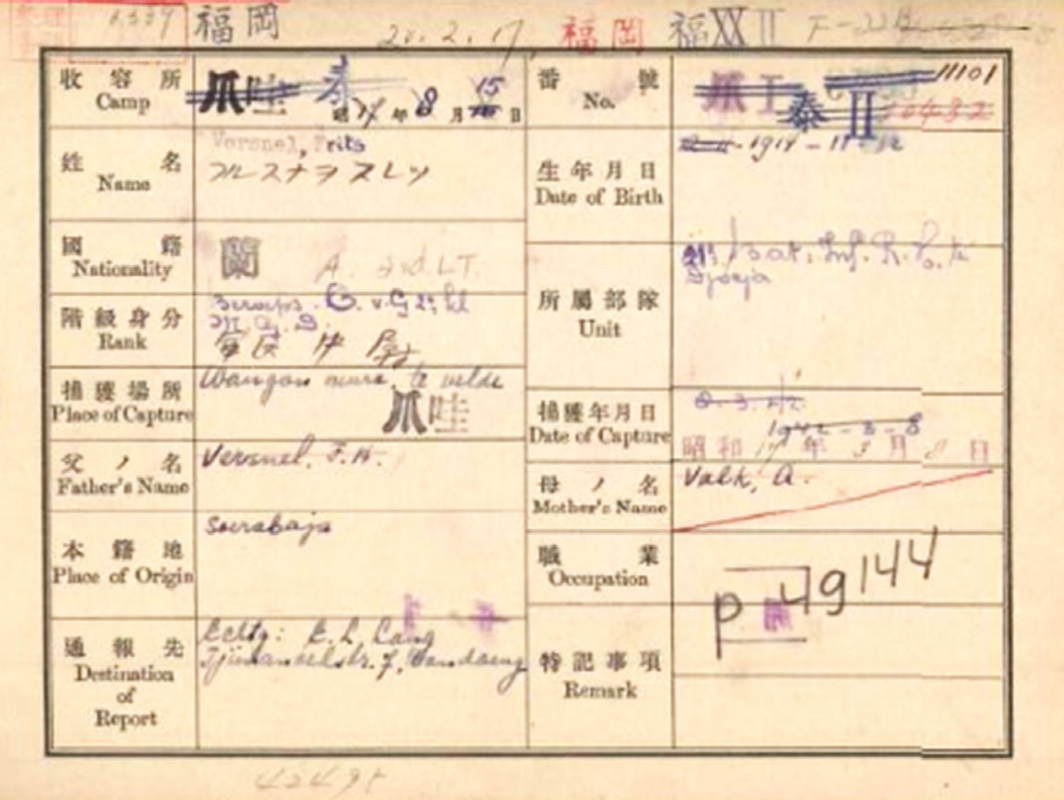
Frits Versnel’s Japanese internment card. Dutch National Archives, *op. cit.* (note 33), public domain.

**Figure 5.**
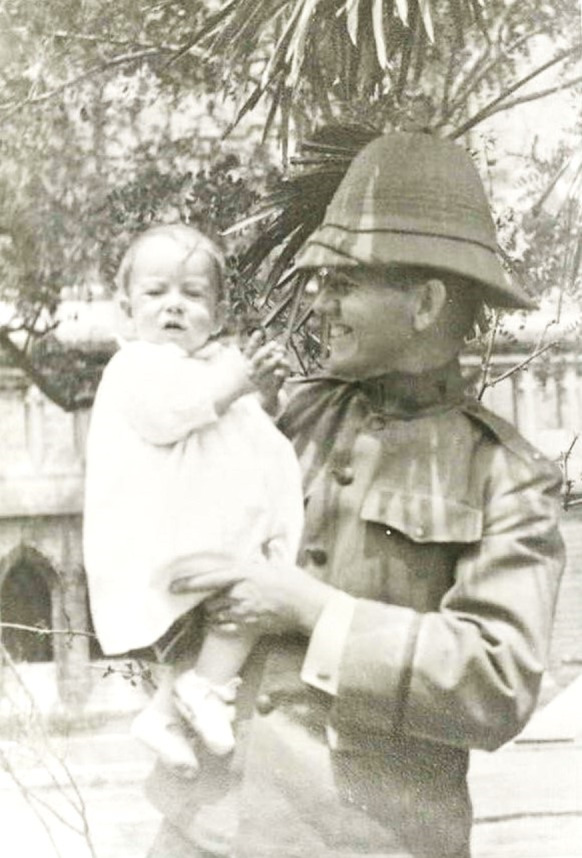
Wibb Cooper and baby son in the Philippines, about 1913. Ancestry.co.uk, Cooper family, with permission.

Many doctors had connections with the region. Their Japanese PoW cards show that four Dutch doctors gave Indonesian contact addresses and one (Versnel) was born in the Dutch East Indies (Figure 4) which implies not only that they were residents, but also that their wives and children may have been among the many civilians interned by the Japanese.^33^ The four IMS doctors had worked in India (which then included modern Pakistan and Bangladesh) since the 1930s. This hybrid civil and military service originated with the East India Company and would form the nucleus of medical research and public health institutions after Independence.^34^ Francis Allinson, one of twin surgeon brothers who served in the IMS, would become Professor of Surgery in Dacca after the war.^35^ Several American doctors had served in the military in the Philippines and would return after the war (Figure 5).

The search threw up incidental family information. Australian Colonel Derham’s son Thomas (1918–79), a private in the 2/9^th^ Field Ambulance, was in the same camps as his father. Australian Dr Patrick O’Donnell’s brother Ian (1905–84), an engineer officer, was imprisoned with him in Heito. Their sister Alice (1901–43) was one of the AIF nurses killed when the hospital ship *Centaur* was sunk in 1943.^36^ British Brigadier Stringer’s wife, Olga (1890–1942), a volunteer nursing assistant, also died at sea on the *Tanjong Penang* in one of the attempts by nurses to escape from Singapore in February 1942.^37^

## First-person accounts

First-person accounts are necessarily subjective and the influential military historian, John Keegan’s, statement fifty years ago that allowing combatants ‘to speak for themselves’ is ‘an essential ingredient of battle narrative’ was innovative at the time.^38^ Keegan emphasised that contemporaneous impressions were the most valuable and, recently, Frances Houghton, in her study of veterans’ memoirs, reiterated the *caveat* that accounts written many years later are written and published in a social and cultural context with a particular narrative of the war.^39^

Paper was scarce in the camps and the Japanese monitored and sometimes confiscated personal diaries. Dr John Glover wrote that the Japanese ‘were assiduous in preventing the collection of records and in destroying such scanty manuscripts as it was possible to write.’^40^ Despite this censorship, the search identified several relevant records deposited in British and online archives. Other medically related Taiwan material may well survive.^41^

The diary kept by Canadian Ben Wheeler is a good example.^42^ His daughter, Anne Wheeler, is a film director and combined her father’s diary with oral history from his colleagues to make a drama-documentary, *A War Story.* ^43^ One of her sources was Dr Peter Seed who deposited his interview transcript in the Imperial War Museum along with notebook entries, letters and drawings.^44^ Before the Wheeler files were closed his diary was quoted by others.^45^

The Blair papers are another important source, a large archive spread across four collections. George Blair’s experience was unusual in that he spent all his Taiwanese captivity in one camp, Taihoku, where for over a year he was the only doctor.^46^ His papers mainly comprise family letters and factual clinical records but information about camp conditions can be inferred, and the Wellcome and Royal Army Medical Corps (RAMC) Headquarters Collections include drafts of a thesis.^47^

One of the most interesting contemporaneous records is that by Gunner John Clement, who developed tuberculosis, giving him a patient’s perspective on a succession of doctors in five camps in Taiwan.^48^ He was a commercial artist before the war and illustrated his detailed and meticulous diaries with profuse coloured crayon sketches. The tone of the drawings is often humorous, which may have protected them from destruction.

There is other material written immediately after the war, while memories were fresh. Drs Wheeler, Blair and Lewis accompanied the sickest PoWs on the New Zealand hospital ship *Maunganui* after they were liberated and each contributed a short, factual essay to the ship’s newsletter, *What Knots.* ^49^ Several doctors sent affidavits to the British War Crimes Trials in Hong Kong in 1947 and 1948 in which there were 95 charges of 123 individuals of which 25 charges related to Taiwan.^50^ Two doctoral theses (by Blair and Glover) were written for British universities immediately after the war.^51^ John Bennet’s scholarly contributions on malnutrition include a substantial *Introduction* to the Medical Research Council’s *Special Report No. 274* in 1951.^52^ Wibb Cooper wrote an official report on the US Army Medical Corps’ work in captivity.^53^

Personal memoirs were published later, and this search identified six, all by Americans. The first to appear was Braddock’s in 1961, in which Taiwan was only one episode in a life which included teaching in Japan, Serbia in the First World War, a brief spell in the RAMC, a Mexican oil company, a mining village in Nevada and the US Veterans Administration. Although then aged over 50, he rejoined the US Army in the Second World War, was posted to the Philippines, went to Taiwan with other senior officers on the *Lima Maru* and was eventually liberated in Manchuria by US paratroopers and Russian troops. Three memoirs appeared in the 1970s: Goodman’s in 1972, Gaskill’s in 1975, and Poweleit’s in 1976. In the 1990s, accounts were published by Coone (1992) and Donovan (1998).^54^

This article does not aim to produce an exhaustive bibliography but there are several relevant memoirs by non-medical PoWs.^55^ Accounts of interest include Brougher’s edited diary, Braly’s detailed memoir published immediately after the war, Kent Hughes’ epic poem, the short but beautifully written account of Shirakawa camp by David Piper, the future art historian, and Padre Fred Stallard’s collection of sketches, also from Shirakawa.^56^ As a Dutch curator wrote, ‘Drawings are of major importance because there are hardly any photographs.’^57^

## Heito, Taichu, Kinkaseki, and Taihoku camps

There were four principal labour camps in Taiwan, each housing about 400–500, where prisoners worked for public authorities or commercial companies. At Heito they cleared ground and moved rocks in order to plant sugar cane and also worked in a sugar cane factory. The camp was closed in March 1945 after Allied bombing. At Taichu (River Valley, Wall) camp, PoWs dug a flood diversion channel and flooding closed the camp in July 1944. Kinkaseki, the copper-mining camp, was active until May 1945. Also in the north was Taihoku (Bamboo, Paradise) camp, near Japanese headquarters in Taihoku city, where the men constructed roads and a ‘Victory Park’ and worked in a train and bus repair shop.

**Figure 6.**
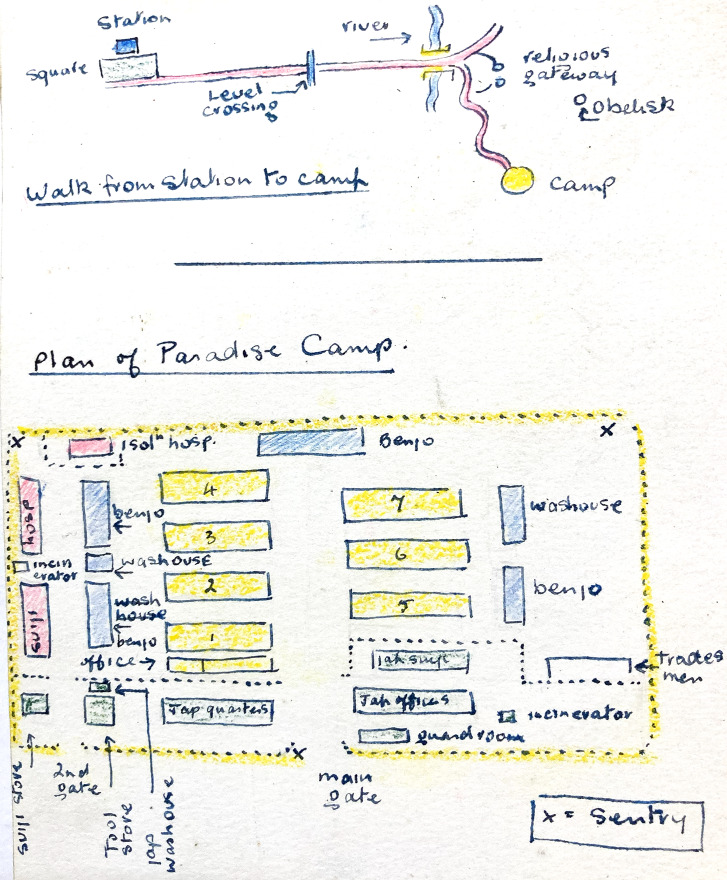
Plan of Paradise (Taihoku) camp. *Benjo* = latrine. Clement, *op. cit.* (note 48), with permission.

**Figure 7.**
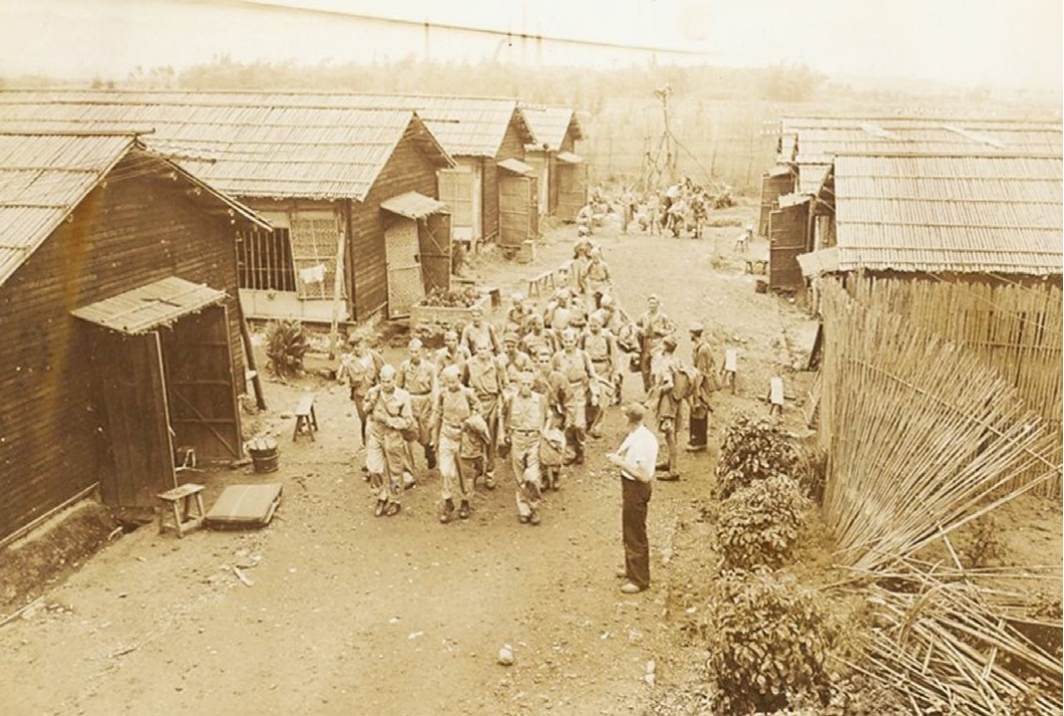
Huts in Taihoku camp at liberation. USS *Block Island* Association, *Formosa POW Rescue*, *op. cit.* (note 59), with permission. No photographer credited.

Kinkaseki is the camp most frequently associated with Taiwan, perhaps because the dangerous labour in an underground copper mine, coupled with an exhausting climb between camp and mine at the beginning and end of each work shift, corresponds most closely to the popular image of captivity under the Japanese. But, medically, Taihoku is the best-documented camp because of Blair’s surviving files. In Piper’s opinion, Taihoku was ‘a tight hard and claustrophobic camp’.^58^ We know what it looked like from sketches, photographs from the time of liberation,^59^ and War Crimes Trial testimony: a collection of bamboo huts surrounded by a bamboo fence. In the accommodation huts, men slept on a continuous shelf down each long side, and a line of narrow tables down the centre was used for eating and other activities. Each was partitioned into four messes, three with twenty-four men each and one for twelve. The camp also contained latrines, a cookhouse, stores, a hospital, and isolation huts. Clement’s coloured plan is in Figure 6 and a view at liberation is in Figure 7. The Imperial War Museum art collection includes a series of watercolours by James Morris, a British war artist who accompanied the liberation force.^60^

**Figure 8.**
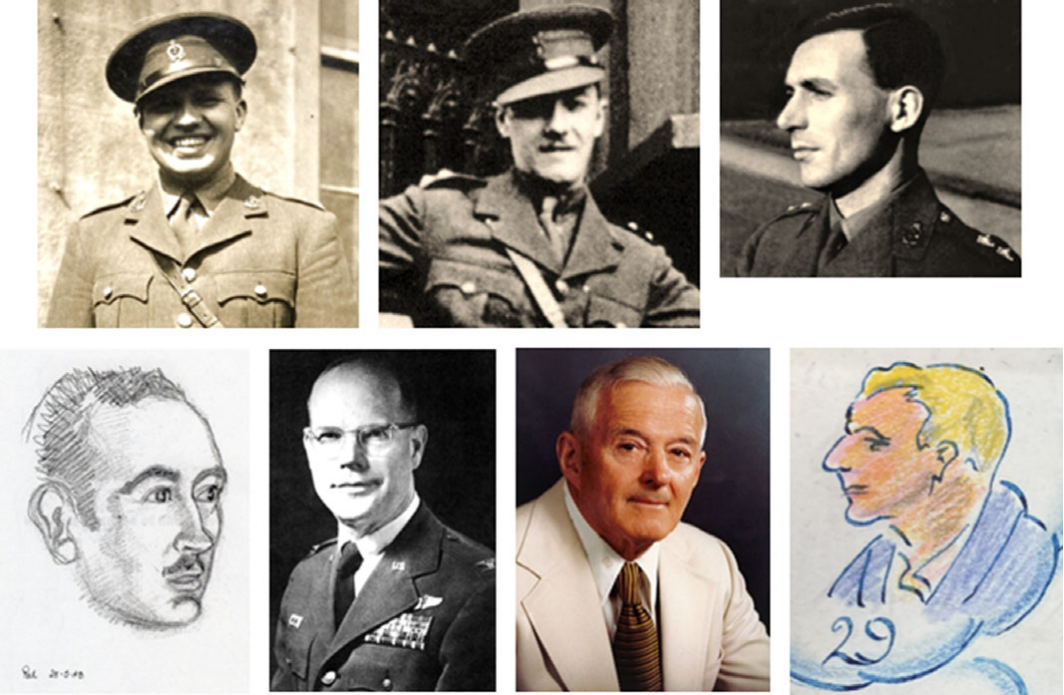
Doctors stationed at the four work camps, 1942–45. Top row from left: Blair (Wellcome PP/GBL, *op. cit.* [note 47]), Wheeler, Seed (powtaiwan.org, *op. cit.* [note 27]). Bottom row from left: O’Donnell (Stallard, *op. cit.* [note 21]), Coone (powtaiwan.org, *op. cit.* [note 27]), Allinson (RCSEng *op. cit.* [note 35]), Glover (Clement, *op. cit.* [note 48]). All are either public domain or with permission. Grant is missing.

For most of the war, these four camps with their shifting population of almost 2,000 were staffed by only seven doctors: six from the British forces (four RAMC and two IMS) and one Australian (Figure 8). Drs Grant and O’Donnell arrived in Heito in August 1942 with the ‘Special Party’ of senior figures from Singapore,^61^ Drs Allinson and Glover worked in Taichu, Drs Wheeler, Seed and Blair arrived in November 1942 on the *England Maru*’s second voyage from Singapore, accompanying 1,100 men from three artillery regiments in ‘Overseas Party ‘Z’, and covered Kinkaseki and Taihoku.^62^ In November 1944 sixteen American doctors arrived from Manila, survivors of the ‘hellship’ *Hokusen Maru*, and added temporary medical staffing in several camps. One of them, ‘Herb’ Coone, came to Taihoku in February 1945 with a party of sick Americans and left for Oka in June. He was followed in March 1945 by Grant and O’Donnell from Heito. Blair wrote, ‘as a result of these moves I have taken over the Hospital and BB patients, O’Donnell continues to do the Fevers, while Grant does the surgical side, including the town party.’^63^ Transfers of doctors are discussed more fully below.

Doctors reported to the senior Allied officer and were involved with the rare visits by International Red Cross representatives. Interactions with the Japanese commander were not always constructive; doctors were sometimes beaten (‘the clouting’ according to Seed) and Piper recalled the commander at Shirakawa telling ‘the doctors they should work harder – there were too many people dying’ and that ‘walking barefoot on dewy grass’ was good for beri beri.^64^ Hurst records that, unusually, the doctors took over as joint commanding officers (COs) at Heito in 1944 after the other officers were transferred. Most unusually for Japanese camps, Heito also contained a secret radio until 1943, smuggled in from Changi.^65^ Hurst’s oral history documents that Dr Grant took it over after its original builder left, a dangerous responsibility.^66^

Trained medical orderlies provided nursing care and the doctors spoke highly of these men. Other PoWs also volunteered. For example, Clement, while a long-stay hospital patient, nursed a man with a leg fracture who was immobilised by traction and Dr Blair notes the names of several officers and other ranks who volunteered in the Taihoku sick bay or hospital. The military chaplains were expected to work with the medical team alongside their pastoral duties.^67^ Anglican Tom Pugh (1903–80) visited the Taihoku hospital regularly and also became George Blair’s messmate. Jesuit Richard Kennedy (1906–86) ran the diarrhoea ward at Kinkaseki for a time, as well as befriending Dr Peter Seed, a fellow Catholic.

**Figure 9.**
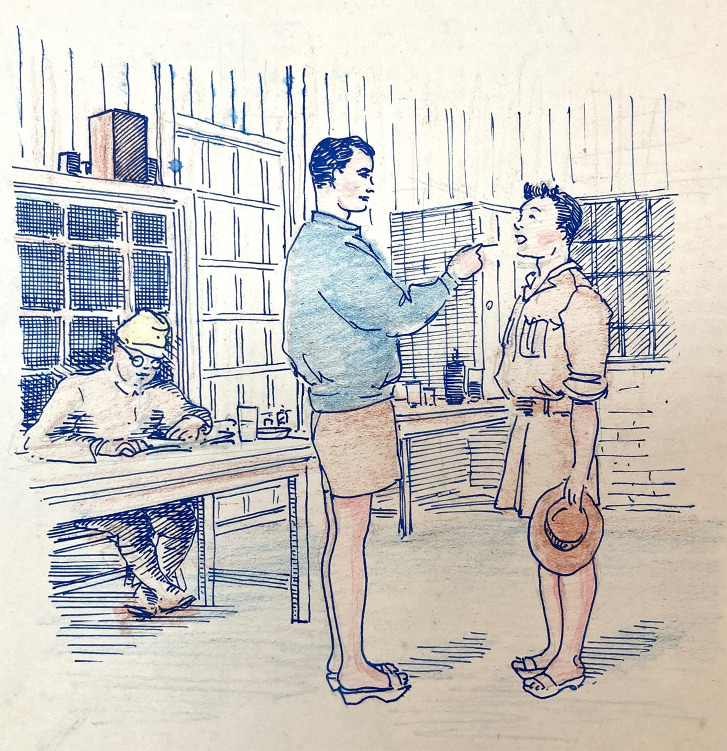
Sick-bay consultation at Kinkaseki. Seed, *op. cit.* (note 44), public domain.

The daily timetable was similar across the camps. Blair notes a sick parade after each of the three camp meals, the one after lunch including a weekly review of beri beri cases.^68^ He did morning and evening ward rounds and two minor operations and dressings sessions per day and recorded draining abscesses and treating foreign body injuries to the eyes of train and bus repair workers. The Japanese medical orderly attended after the morning sick parade and Peter Seed’s papers include a drawing, clearly sanitised, of a sick bay consultation (Figure 9).

## Medical practice

Japanese military culture meant there was a constant background of seemingly casual violence in slappings and worse beatings, but their war crimes were, largely, medical and the PoW experience was of starvation, malnutrition, infections and infestations. The cookhouse and latrines dominate diaries. Meals were rice-based and deficient in protein, calories and several vitamins.^69^ The doctors were involved in overseeing aspects of diet, encouraging the eating of rice ‘polishings’ (husks), for example, to increase the intake of B vitamins. Most prisoners exhibited oedema caused by vitamin B deficiency, possibly combined with protein deficiency. Men displayed considerable ingenuity in buying or bartering additional supplies from local people and in foraging for food. Clement, for example, describes gathering bamboo shoots and catching snakes and snails: ‘got 3 snails also…baked in the embers…burnt them but will eat anything’, ‘Nearest approach to fish and chips I’ve had for a long time.’ For the senior officers, snails were ‘Karenko clams’.^70^

**Figure 10.**
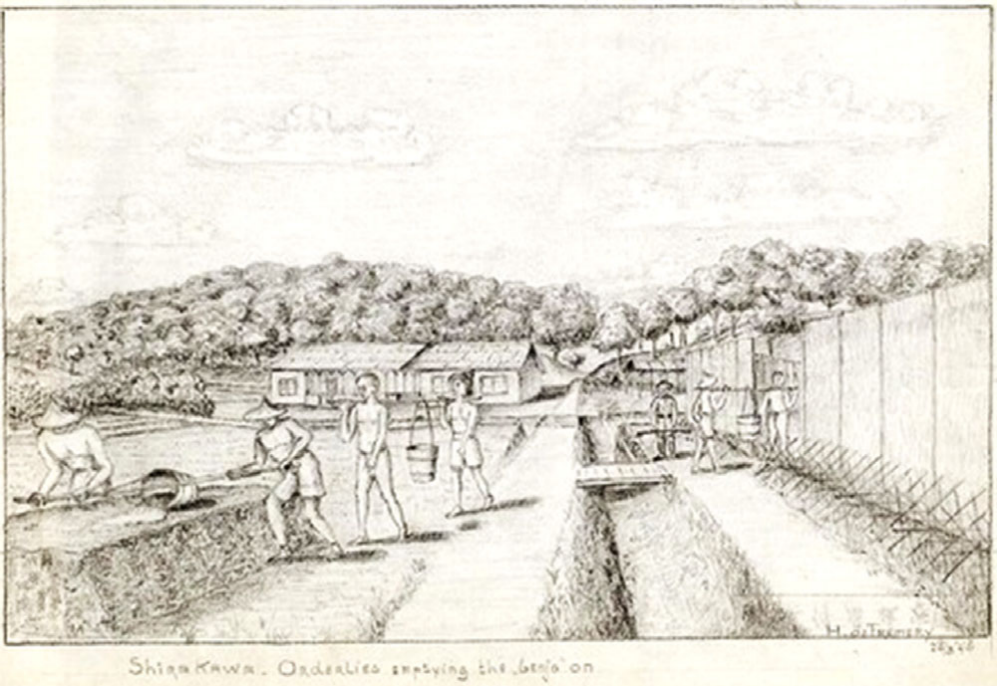
Carrying *benjo* buckets to fertilise the fields, Shirakawa. De Fremery, *op. cit.* (note 97), with permission.

The Japanese organised immunisation campaigns and enforced isolation arrangements for infectious diseases but latrines were usually of the pit type, close to the cookhouse, emptied by hand, and the contents used to fertilise fields (Figure 10). Insanitary conditions led to repeated outbreaks of diarrhoeal disease and infestations with intestinal worms that could not be eradicated. Skin conditions could lead to ulcerated sores. Malaria does not appear to have been as much of a problem in Taiwan as it was in the jungle camps in Thailand and Burma, but there were recurrences from infections acquired before capture and, in parts of Taiwan, mosquitoes were prevalent.

Prisoners were regularly weighed and all lost weight, even the most senior officers. Blair’s notes from August and October 1943 record camp averages of only 55 and 53 kg and PoW Lionel Haylor’s diary states that his usual weight was 85.7 kg but fell to its lowest in 1945, between 47.2 and 50.8 kg.^71^ Dispersal decisions often depended on weight: heavier PoWs were deemed capable of heavier work and ‘thin man’ parties were transferred from work camps, such as Kinkaseki, to other camps, such as Shirakawa. Food, work, weight and illness were closely interrelated. It was Japanese policy that work was reflected in pay and rations, a policy which had its greatest impact on the sick. If a camp’s proportion of sick rose, less food was supplied, but most camps shared food equally and would supplement hospital rations when they could, which meant a deficient diet for all. Prisoners were placed into four groups: fit for heavy work, fit for light work, sick in camp, and sick in hospital. These categories were strictly enforced, and each camp had only a limited allocation of those tokens which denoted sickness. On a daily basis, the doctors mediated between men who were unable to work and Japanese medical orderlies who required a man to be visibly sick.

Camp doctors had few resources. They carried enough medical kit and stores from their camp of embarkation to cover the journey to Taiwan, ‘two Regimental Medical Panniers’ in Blair’s words,^72^ but received little in the way of replacements. When gauze dressings and bandages had rotted after repeated rewashings, ‘I [Blair] used cellophane squares from the inside of Japanese cigarette packets. … When the Japanese no longer packed their cigarettes in cellophane…I had to use ordinary newspaper…’.^73^ An ‘extraction of cinchona bark’ was prepared at Heito, ‘a revolting potion. It kept the malaria at bay.’^74^ Clement records a medical orderly boiling up ‘dog ends’ in Inrin as a topical application for pellagra. There are records of Japanese-supplied drugs such as aspirin, quinine and a vitamin B preparation but ‘in general the Orderly would not give more than three days’ supply at once for any one man’ and the doctors had to ration these scarce medications.^75^ They performed paracenteses to draw off peritoneal fluid to relieve abdominal distension in beri beri cases. For example, fourteen pints were recorded by Blair on 27 August 1943 and ten from a different patient on the following day.^76^ He used Southey’s tubes (small cannulae) to drain fluid from oedematous ankles and gave small blood transfusions (the donor rewarded with an egg), procedures also carried out by other doctors elsewhere.

Despite these conditions, the doctors kept to a routine and made careful records. The Blair archive, for example, contains a large hard-backed book, originally designed for recording stores required for naval construction and presumably salvaged from the naval dockyard in Singapore, which became the Taihoku hospital admissions book. Small notebooks supplied by the Japanese record weights, administrative data and case histories. Between the pages are loose and evocative scraps of paper and card which contain surviving temperature charts, fluid balance charts, prescriptions and other temporary notes.

## Patterns of illness and mortality in labour camps

The pattern of illness and death in Taiwan was broadly similar to the pattern elsewhere and technical medical details are not recapitulated here.^77^ In vivid lay language, Piper wrote ‘I see back after back, wing-boned, vallied and shrunken in the skeleton. The sharp nobbled ridge of the back-bones… Ankles swollen like bladders, fold on the joints like a pillow folded, and the odd sprawl of toes from the shapeless balloon. Apathy of the eyes. Skin parchment texture, ingrained with grime, and blotched with sores and eruptions.’^78^ One of Glover’s strongest memories was ‘the sound of noisy watery defaecation in the latrines; of tell-tale trails of liquid faeces on the ground…’^79^ Blair remembered that ‘hunger was always predominant’, ‘many men had a mild degree of oedema throughout their captivity’, ‘worms were continually troublesome [after June 1943]’, ‘malaria was common’, and ‘skin diseases were constantly present’.^80^ Interviewed in 1977, Seed remembered dying men, ‘I was the last human being they would have contact with in this squalid death at the other end of the world from their homeland.’

**Figure 11.**
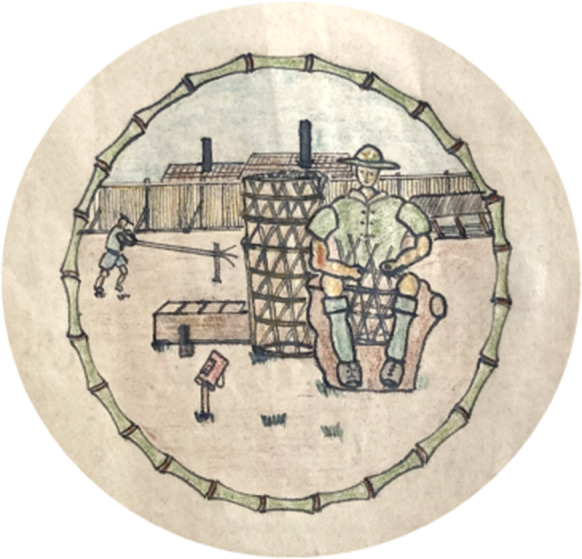
Light duties making a bamboo basket, Taihoku. Detail from 1944 Christmas card. RAMC HQ, *op. cit.* (note 47), public domain.

The War Crimes Trial files include a snapshot for Taihoku on 21 September 1943 when ‘105 men (over twenty per cent of the camp strength) were sick: 18 in hospital, 34 allowed to lie down in their huts, 46 carrying out light duties in camp, and seven who had reported sick but were not yet categorised. Of the sick, 35 had beri beri, 28 had disorders such as fevers, jaundice and diarrhoea and 42 had skin conditions including ulcers and boils.’^81^ A hand-drawn greetings card illustrates the bamboo basket-making work which the sick carried out at Taihoku from October 1943 (Figure 11).^82^ The Blair archive contains records of sick-bay attendances for less serious illnesses; we know, for example, that the only illness Padre Tom Pugh suffered was an episode of dengue, and he was not hospitalised.

**Figure 12.**
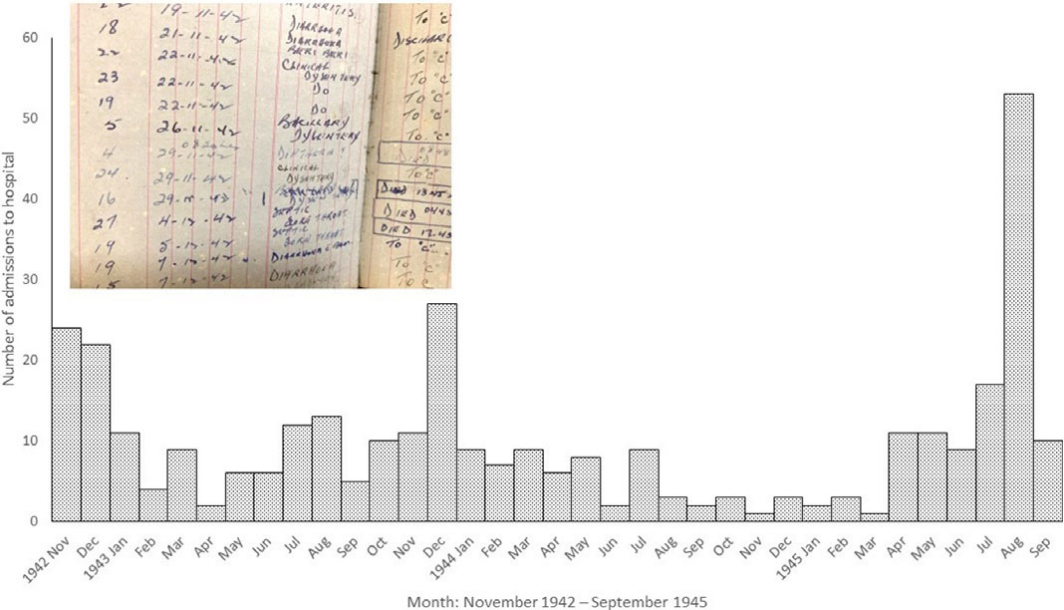
Monthly admissions, Taihoku hospital. Extracted from data in Museum of Military Medicine, *op. cit.* (note 47), public domain.

The Taihoku hospital admissions book provides a complete sequence of admissions and in-hospital deaths from one camp. Figure 12 shows the monthly hospital admissions. Most were for diarrhoeal diseases or beri beri, in its various forms. The peak at the beginning of Taiwanese captivity is related to the outbreaks of diarrhoeal disease and diphtheria that started on the ‘hellship’ *England Maru.* An anonymised extract in Ben Wheeler’s handwriting from the book’s early pages is also shown in Figure 12 and illustrates the precision the doctors attempted despite their lack of diagnostic resources: he distinguishes between ‘bacillary dysentery’, ‘clinical dysentery’ and ‘diarrhoea’. The peak at the end of the war occurred because the hospital was extended to take sick men from across the island ready for repatriation. At this stage, men were dying of starvation and diagnoses simply of ‘anorexia’ and ‘debility’ were recorded.

**Figure 13.**
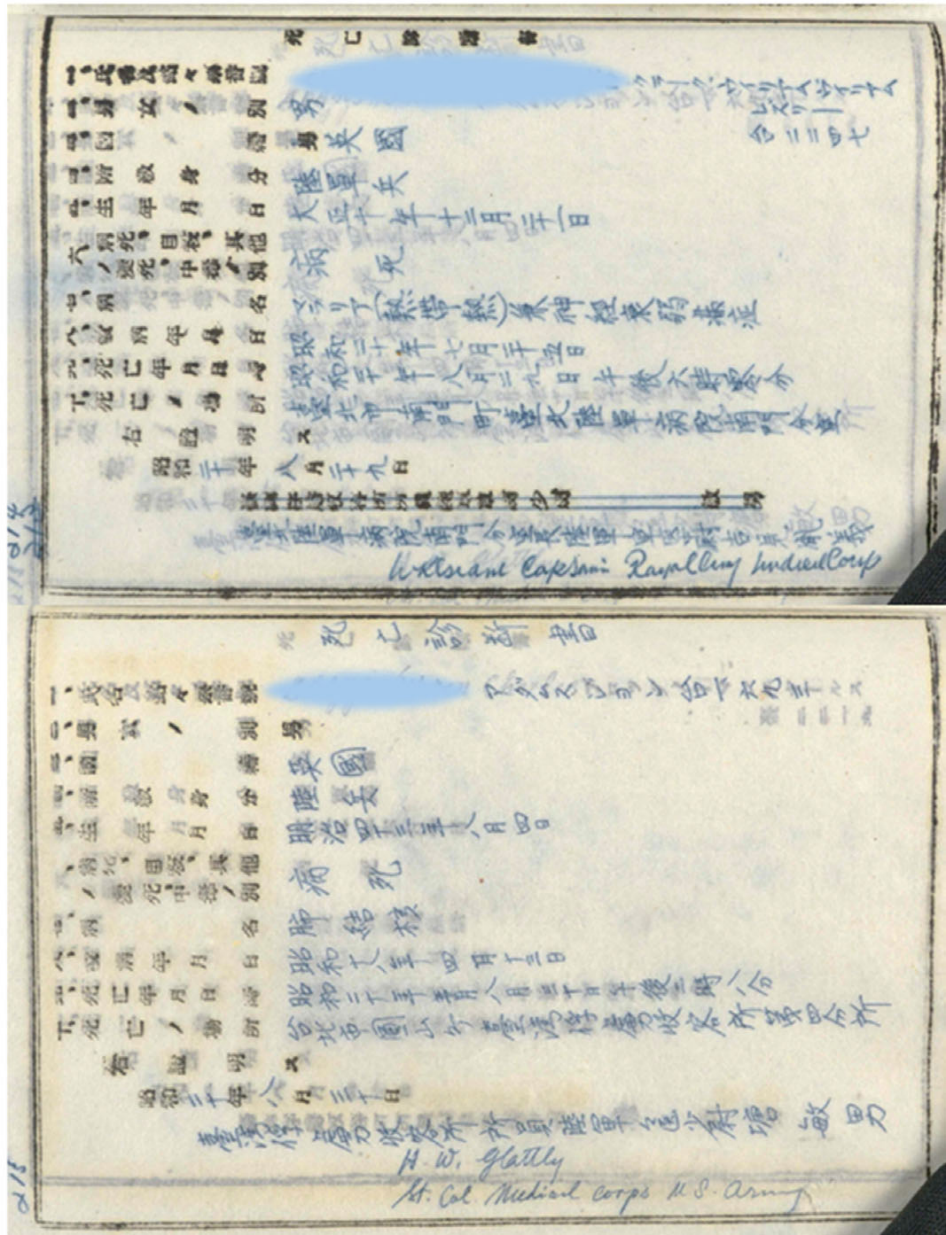
Japanese PoW death certificates (anonymised). Signed by Grant (top) and Glattly (bottom). TNA: WO/361/1475, *op. cit.* (note 83), public domain.

**Figure 14.**
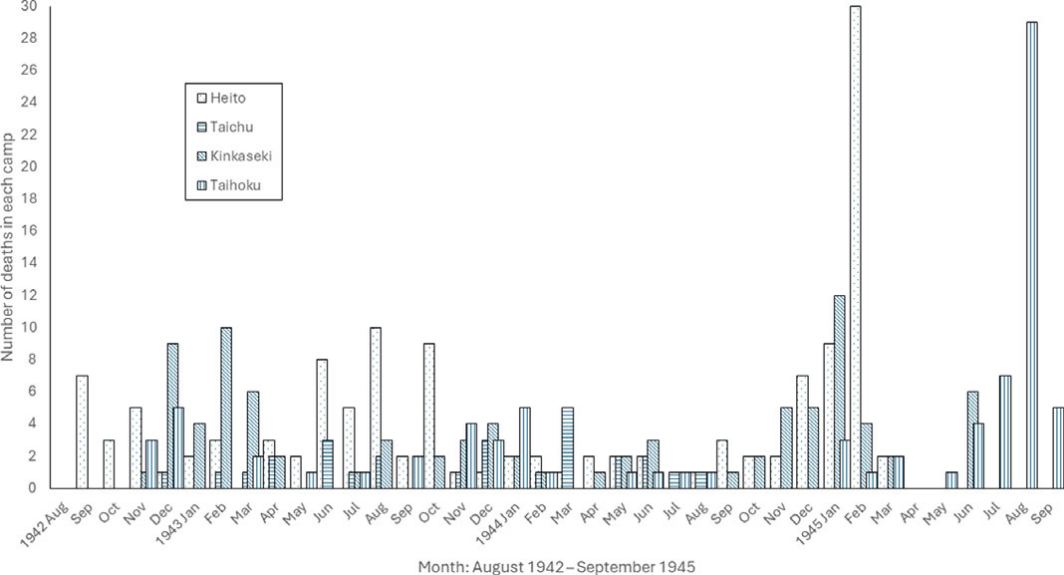
Monthly deaths in the four labour camps. Extracted from data in WO/361/1475, 1757 and 1758, *op. cit.* (notes 83 and 84), public domain.

Mortality data were also recorded in as precise terms as possible and the Japanese authorities required a death certificate signed by an Allied doctor (Figure 13).^83^ At liberation, Allinson and Blair gave lists of deaths under their care to MI9, the British agency responsible for interviewing repatriated or escaped PoWs.^84^ Lists of deaths at Heito and Kinkaseki,^85^ with their causes, are also preserved in The National Archives and these four sources provide information on the deaths of 330 prisoners from the four principal labour camps in Taiwan (Figure 14).^86^ This number is an underestimate of the total. There were at least twenty-two additional deaths which occurred at sea or in other camps and deaths elsewhere in Taiwan are not included in Figure 14.

The first deaths in this series occurred in September 1942 during an outbreak of dysentery at Heito, the first camp to open. One of the doctors, William Kennedy, was among the seven dead. Heito’s sugar cane plantations were created from malarious swamps. Of the 129 recorded deaths, causes are available for 111 and, of these, twenty-three are listed as due to malaria alone, twenty-six from malaria and beri beri combined, and thirteen from beri beri alone, over half of the total (62/111, 56%). A further thirteen included malaria or beri beri as one of several causes of death (75/111, 68%). In February 1945, an Allied air raid killed more than twenty-five prisoners and injured others, leading to the camp’s closure. Blair noted that some men still had unhealed wounds from the bombing when they arrived at Taihoku.^87^

At Taichu, a flood closed the camp after less than two years of operation. Allinson’s list of twenty-six deaths is unusual in the prominence it gives to pellagra, mentioned in almost half of the deaths (12/26, 46%). Deficiency of niacin (vitamin B3) causes pellagra, which gives a characteristic roughened appearance to sun-exposed skin, as well as diarrhoea and cerebral symptoms. He gave it as the only cause of death in two, in nine combined with beri beri and, in one, with beri beri and possible meningitis. The prominence of pellagra raises the usual questions about the subjectivity of death certification: it is possible that the diet at Taichu was more deficient in niacin than the diet in other camps, where pellagra is mentioned as a cause of death only rarely, but also possible that Allinson diagnosed it more frequently than other doctors, either because he was more familiar than they were with its manifestations (in the IMS since 1933)^88^ or was more likely to attribute lethality to it. As in other camps, beri beri was a major cause of death, listed alone for one death and in combination with other causes for eighteen (19/26, 73%). Dysentery, malaria, meningitis and scabies are also mentioned.

At the Kinkaseki copper mining camp, Seed was joined by Wheeler in August 1943. In March 1945, Seed and Wheeler were transferred to Shirakawa hospital with a group of sick men. Unsurprisingly for a mine worked by slave labour, eight of the ninety-one deaths were due to injury, for example, crush injuries and fractured skulls. There were also non-fatal mining accidents; Seed remembered skull fractures and Blair recorded ‘several old mine injuries’ which would not heal in men transferred to Taihoku.^89^ Illustrating the semantic aspects of death certification, the Kinkaseki list uses the terms ‘colitis’ and ‘enteritis’ instead of distinguishing between types of diarrhoeal disease, possibly at the request of the Japanese. These terms appear for over half of the deaths (48/91, 53%). As in other camps, beri beri was a prominent cause of death, the sole cause of twenty deaths, and at least one cause of almost two-thirds of deaths (57/91, 63%). Respiratory illnesses were noted for twelve deaths. Perhaps because of the camp’s altitude, malaria was mentioned for only one death.

Blair was at Taihoku for the whole of its operation, working under Wheeler for the first nine months, then alone until other doctors joined him towards the end of the war. He assembled a list of eighty-three deaths (eighty-six if deaths attributable to transport on the *England Maru* are included). Beri beri was the most common cause, the sole cause in twenty-nine, and one of several in a further twenty-six, two-thirds of all deaths (55/83, 66%). In November 1944, Blair wrote, ‘I have been working on an essay on beriberi but as soon as one argument is advanced, another, diametrically opposed, presents itself!’^90^ He had started this project with Wheeler in February 1943 and would explore the topic further after the war.^91^ A diarrhoeal disease was a cause of almost one-fifth of deaths (16/83, 19%). An outbreak of ‘fevers’ mentioned by Wheeler as starting on the ‘hellship’ led to five deaths probably from diphtheria.^92^ Tragically, Allied air drops of canisters of food and other supplies after the Japanese surrender caused many injuries and two deaths and contributed to a third, one American, one British and one Dutch.

## Senior officers and their camps

An unusual feature of the camps in Taiwan was their use to imprison military commanders, colonial governors, and other senior civilian officials. The first group to arrive in Taiwan, in August 1942, was a party of about 130 American officers with about fifty enlisted men from Manila, in *Nagara Maru*, known to its passengers as ‘Stinko Maru’.^93^ They were followed two weeks later by the *England Maru* on its first voyage between Singapore and Taiwan carrying the ‘Special Party’ of 400 from Singapore, senior figures with their batmen (soldier servants), in convoy with the *Fukkai Maru.* ^94^ Other senior officers arrived from Hong Kong and Java. In rhyming couplets, Kent Hughes lamented that ‘…many a lesser rank could scarce but laugh/ To see a general, stripped down to his skin,/ Collecting water in an old jam tin/ …Gone was vain pride, false modesty, and rank,/ When everybody sweated, gasped and stank.’^95^ They went first to Karenko then, in June 1943, inland to Shirakawa. In October 1944 they were transported further north to Japan, Korea and Manchuria, away from Allied bombing raids. Shirakawa became a *de facto* hospital camp.

To illustrate the seniority of these men, Lieutenant-General Arthur Percival (1887–1966), the British military commander in Malaya, came from Singapore with his civilian opposite number, Sir Shenton Thomas (1879–1962), Governor of the Straits Settlements. Major-General Merton Beckwith-Smith (1890–1942), CO of the British 18^th^ Division, was also captured in Singapore and became the most senior British officer to die in captivity; he died of diphtheria in Karenko.^96^ The CO of the Royal Netherlands Indies Army, Major-General Hans de Fremery (1875–1972) was captured in Indonesia and made detailed pencil drawings of camp life, reproduced in several memoirs.^97^ From Corregidor came General Jonathan Wainwright (1883–1953), who became CO of the American forces in the Philippines after General Douglas MacArthur (1880–1964) controversially left for Australia in March 1942.

The strangeness of the picture of so many senior figures from different countries imprisoned together has appealed to surprisingly few commentators, although Mark Felton and Hamish Ion have published accounts.^98^ Their average age was in the mid-fifties, and many were at the peak of a military career that started in the First World War. They were housed by nationality and Felton and Ion describe a wary cooperation, with tensions which Ion attributes partly to cultural diversity but largely to different political situations. The British Empire was in decline and the humiliating failure of the Malayan campaign had led to the ‘worst disaster’ of the fall of Singapore.^99^ In contrast, the Americans had surrendered last and were now the best-resourced of the Allies, looking forward to post-war global dominance. Furthermore, as several commentators observe, its internment of over 100,000 Japanese citizens and Japanese-Americans gave the US unique leverage in negotiations with Japan.

**Figure 15.**
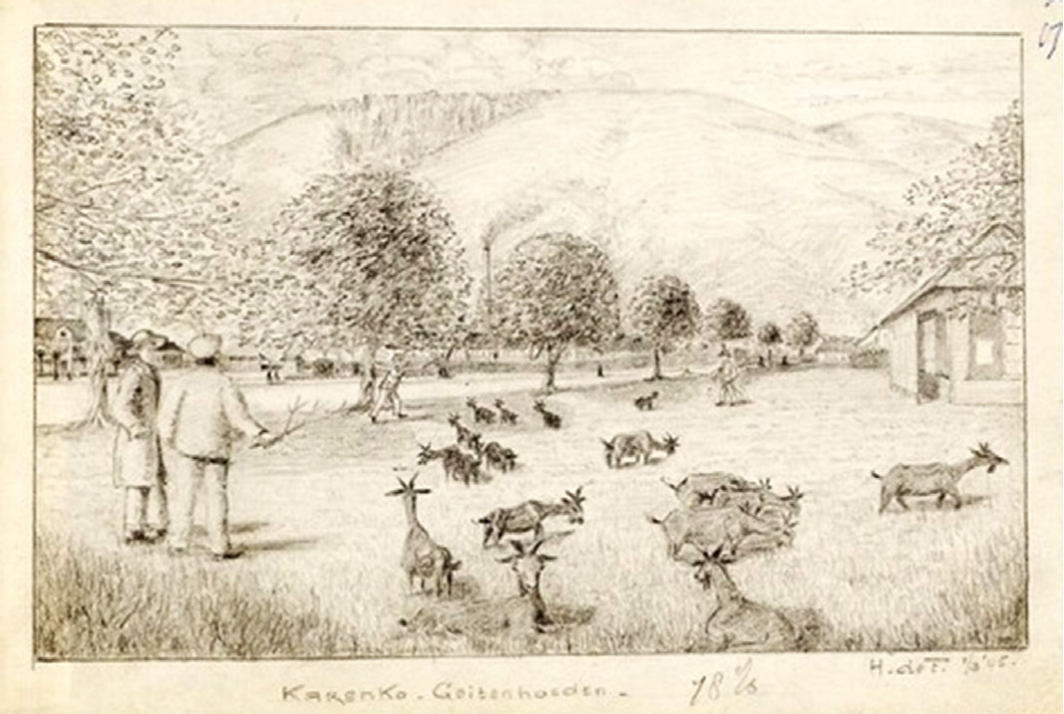
Senior officers herding goats at Karenko. De Fremery, *op. cit.* (note 97), with permission.

The Japanese sought to demean these senior officers and would not allow indicators of rank. Like other PoWs, they were expected to grow food and do housekeeping tasks. Figure 15 reproduces one of de Fremery’s drawings of Karenko where ‘It is instructive to watch “top brass” herding goats’.^100^ Nevertheless, they had sufficient leisure to read and play bridge and, in December 1943, the first issue of a magazine, *Raggle Taggle*, was produced in Shirakawa. Some contributions survived the war to be published in 1947 and include humour, anecdotes, poems and cartoons.^101^ Essays on specialised topics include one on differences between American and British English; ‘lack of space prevents a discussion of the ramifications which a humble word like “biscuit” offers.’^102^ Many officers, such as General Percival, spent considerable time discussing with their peers the events leading up to surrender and drafting what would become their post-war reports.^103^

Accommodation and working hours were better than at other camps but, as Wibb Cooper recalled, in captivity they were ‘beaten and disciplined for the least infraction of petty regulations. We were starved… The majority of the group lost from fifty to seventy-five pounds in weight. Some individuals halved their weight. Practically everyone had a nutritional disorder of some kind. Nutritional oedema was the rule.’^104^ Harold Glattly was the senior doctor for much of Shirakawa’s existence as a senior officers’ camp and hospital camp and ‘managed the hospital very well under the most difficult conditions’.^105^ He had survived the Bataan Death March and would become a distinguished figure in the post-war field of prosthetics for amputees.^106^ Senior officers were well-supplied with doctors, relative to population; when about 100 were moved temporarily to Tamazato they were accompanied by two doctors (Derham and Glattly), with medical orderlies.^107^

The seventeen senior doctors were imprisoned only in one or more of the senior officers’ camps, so it is clear that military rank determined their disposition rather than, say, professional specialty. Their pre-surrender roles carried heavy responsibilities and included, for example, Chief Surgeon to the US Asiatic Fleet (Lowman), CO of Canacao US Naval Hospital (Davis), CO US Army No. 2 Hospital (Gillespie), Divisional Director of Medical Services (DDMS) 8^th^ Australian Division (Derham), ADMS 11^th^ Indian and 18^th^ British Divisions (Mitchell and Wardle), CO 10^th^ and 13^th^ Australian General Hospitals (White and Pigdon). The Australian official history recounts that Derham had asked the CO of the AIF in Malaya, Major-General Gordon Bennett (1887–1962) to evacuate the Army nurses from Singapore but Bennett delayed, citing the effect on civilian morale. After it eventually left, the SS *Vyner Brooke* was sunk off Sumatra and survivors who reached Banka Island were machine-gunned by Japanese soldiers as they attempted to surrender. Of sixty-five Australian nurses on board, twelve were killed at sea, twenty-one killed on Banka Island, and thirty-two interned in civilian camps, of whom eight died in captivity.^108^

## Temporary holding camps

Two of the ten temporary camps, Tamazato and Moksak, were used to segregate groups of senior officers in better conditions prior to (rare) visits from the International Red Cross. Another camp was at Takao harbour where many ‘hellships’ docked and at least one death occurred.^109^ Three temporary camps were set up in 1944: Inrin to take PoWs from Taichu after the flood, and the Inrin Temporary and Toroku camps to take survivors of *Hokusen Maru.* Kukutsu and Oka were set up in mid-1945 and several accounts speculate that they were intended as extermination camps, should there be an Allied invasion; after the war, a draft ‘annihilation order’ was found in Taiwan.^110^ Churon and Maruyama camps existed only for a matter of days, set up after the Japanese surrender to house PoWs from across the island before evacuation.

## Transfers of doctors

Multiple transfers were the norm for prisoners of the Japanese. The northward moves made by senior officers are mentioned above. Taihoku had initially housed men from three British artillery regiments and Divisional signals and other staff but, because of repeated transfers, ended the war containing men from fifty-three different British units, as well as Americans, an Australian and a soldier from the Straits Settlement Volunteer Force.^111^ Blair’s static captivity was unusual.

A group of American doctors made a series of transfers in 1944 and 1945. They were captured in the Philippines and had been through several camps before embarking in Manila in September 1944 on the *Hokusen Maru*, known as ‘Horror Maru’ or ‘Benjo Maru’.^112^ Their convoy was depleted by Allied attacks and on arrival in Hong Kong was attacked again before leaving for Taiwan. The total journey duration was 39 days, with the same number of deaths. Robert Gaskill ‘lived in a sea of excrement in the hold’. PoWs were ‘dirty from head to foot, ragged, and swollen with beri-beri’.^113^ Dr Coone lost 36 lbs in weight, despite bloating from oedema, and ‘we were covered with vermin, and filthy’.^114^ Gaskill, the senior doctor, organised a rota of deck visits by groups of twenty but, in the heat, dehydrated men were ‘going insane’ and Raulston ‘was forced to resort to physical force to keep order in his corner.’^115^ After their arrival, they spent several months in Shirakawa, ‘a lovely mountain resort’ with ‘clean and well-ventilated’ barracks where Coone roomed with Schneider, Goodman with Waters, and Poweleit with Donovan and several British officers.^116^ Goodman reports tensions with British officers over food distribution.^117^

After a few months, the Japanese transferred most of the Americans further north, but Dr Goodman was delegated to accompany a group of men with encephalitis to Toroku and later on to Moji and Nagasaki in Japan, where he would in August 1945 hear the ‘indescribable, earth-shaking sound’ of the atomic bomb, followed by an ‘all-encompassing silence’ then ‘a violent hurricane wind’. He was repatriated through the port and saw the ‘rubble and debris’ of the ‘flattened’ city.^118^ Dr Gaskill left Taiwan on the *Melbourne Maru* for Japan, where prisoners worked for the Mitsubishi company in a lead and zinc mine in Sendai, about 300 miles north of Tokyo. Coone, however, was off-loaded from the ship at Kiirun with a group of sick PoWs who, like Goodman’s group, were quarantined with encephalitis. This led to Coone working alongside Blair in Taihoku for four months where, compared with Shirakawa, ‘the men were too weak for sports’. He then took a group to Oka prior to the Japanese surrender.^119^

RAMC Captain Lewis had a particularly harsh transfer experience. The regimental medical officer to the Suffolk Regiment, captured with the 18^th^ Division in Singapore shortly after its arrival, he went from Changi by rail in ‘Letter party ‘R” on 31 October 1942 to the Thai-Burma Railway.^120^ Railway medicine is covered by Gill and Parkes and in several medical memoirs, such as Pavillard’s.^121^ Sergeant Burgess of the Bedfordshire and Hertfordshire Regiment picks up Lewis’ story with a five-day rail journey from Thailand to Singapore in 1944, thirty or more men packed in each goods wagon where ‘our doctors did very good work with the little medical supplies at their command, and on two occasions I saw Dr Lewis (Capt, Suffolk Regt.) extracting teeth with ordinary pliers…’.^122^

In an essay written shortly after liberation, Lewis describes embarking in Singapore on 27 June 1944 on the *Hofuku Maru.* It had engine trouble and took a month to reach Manila where it remained in harbour until 19 September. Ninety-four men died during this almost three months’ ordeal.^123^ Burgess wrote:

dysentery, malaria, yellow jaundice, beri-beri and all manner of diseases were beginning to lift their grisly heads and the conditions were such that they were completely out of the control of the medical officers. Capt. Lewis was consulting the Japs every hour of the day endeavouring to get the conditions altered and had to literally beg for a little extra water for the sick. Needless to say we were not allowed out of the ship’s hold only for the purpose of nature. Even this privilege was denied after a time and the conditions prevailing in our living, sleeping and eating space cannot be conveyed to any but those who have experienced it.

Lewis continued, ‘While in the harbour the Japanese allowed us to evacuate 50 sick to the American hospital at Manila. All of these were so helpless that they could not walk nor do anything for themselves and it was only possible to have some of them removed by giving about 15 blood transfusions.’ Burgess again, ‘Capt. Lewis also performed the almost impossible by carrying out two major operations by the light of hand torches [and] ‘made incisions in both feet [of men with beri beri, to] prevent strangulation of the heart.’

When the ship finally sailed again it had, in Lewis’ account, ‘on board 200 men who were too weak to do anything for themselves. They passed urine and faeces where they lay…’. The convoy was attacked two days later with the loss of twelve ships and about 900 PoWs. Lewis was one of the survivors picked up by interisland patrol vessels which were also attacked by air, killing further PoWs. When they arrived back at Manila ‘all of us practically naked’, they were taken to Bilibid jail, which had become an American PoW hospital. There he met Gaskill, Coone and the other American doctors.

After about a week Lewis and his group of survivors sailed with the American party on another ‘hellship’, the *Hokusen Maru* which was ‘worse than the one we were on previously’. On Taiwan, he went first to Shirakawa where Piper described him as ‘the legendary Captain Lewis, the MO Doctor who removed an appendix in the bottom hold of a Japanese transport, with a razor… Six men to hold the patient down, and a gallery of enchanted Japaneses [*sic*] spectating from the upper walls. His patient was in the first party of survivors to arrive here’.^124^ Lewis later met Blair in Taihoku and sailed with Blair and Wheeler on the hospital ship *Maunganui* to New Zealand. He returned home to Wales and became a rural general practitioner.

## Patients’ perspectives

**Figure 16.**
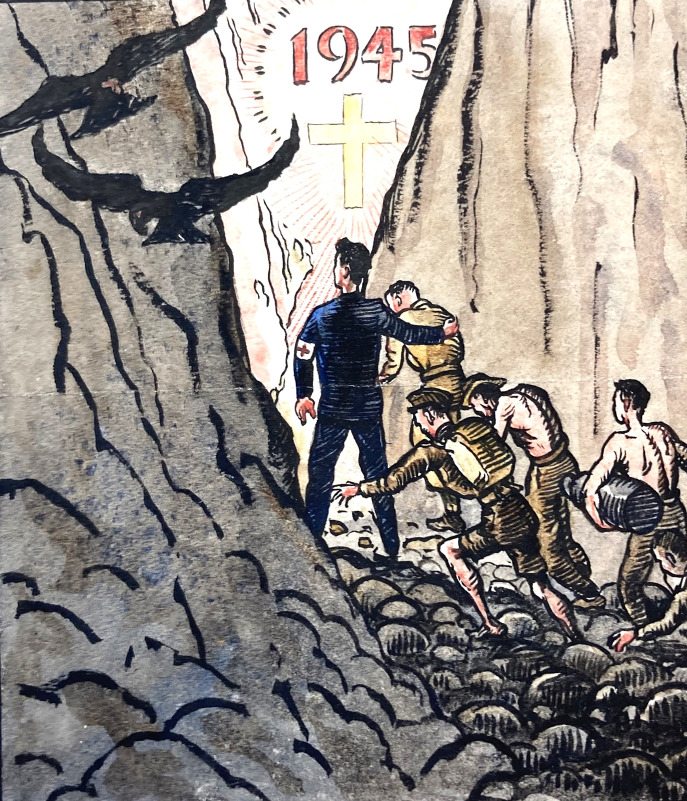
Looking forward to 1945. 1944 Christmas card. Seed, *op. cit.* (note 44), public domain.

Many of the doctors were awarded honours by their country’s government after the war for their work in the camps. It is also clear that the prisoners valued their work. A *Readers Digest* profile of Wheeler used the phrase ‘a man sent from God’ and a similar expression was used for Blair in an appreciation published after his death in 1979 in *The Gunner.* ^125^ A moving tribute survives in Seed’s papers from the ‘squad chiefs’ at Kinkaseki, twenty other ranks who led the work groups. They composed a signed certificate for Wheeler and Seed on behalf of all PoWs to ‘express their high appreciation and deep gratitude’, ‘you have both given of your best and, through you your untiring efforts, many lives have been saved… We realise the limitations of medical aid placed at your disposal.’ A Kinkaseki greetings card with Biblical overtones shows the young Dr Seed as almost a saintly figure leading men towards a better future in 1945 (Figure 16).^126^

**Figure 17.**
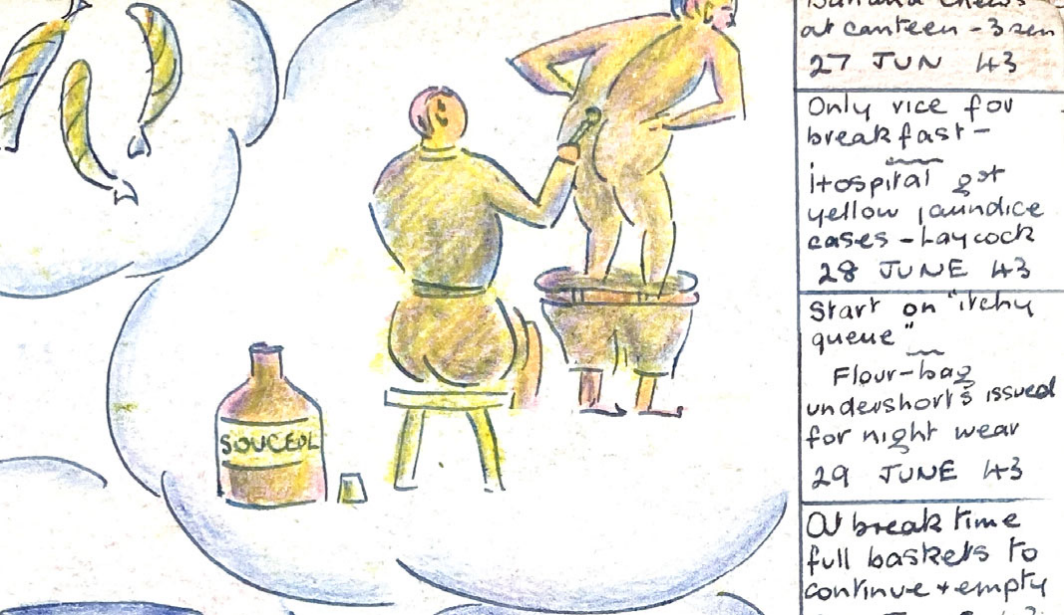
Treatment for ‘prickly itch’. Clement, *op. cit.* (note 48), with permission.

Clement’s illustrated diary gives a more nuanced, often humorous, patient’s perspective and records mundane, but important, interactions with named doctors. He was a private soldier in the 5^th^ Field Regiment, Royal Artillery, to which Blair was attached, and he knew Blair in India, the Malaya campaign, Changi, Number 3 hold of the *England Maru*, and Taihoku. Blair prescribed, successively, an ointment for ‘eyes’, rice polishings for beri beri, baths for ‘prickly itch’ (Figure 17), and ‘hair pomade’ as a treatment for piles. Dr Wheeler and the camp CO ensured that Clement’s spectacles were repaired.

In November 1943, Clement was transferred to Taichu where he met Drs Allinson and Glover. His tuberculosis was confirmed there by a sputum test in January 1944, and he notes also having beri beri, diarrhoea, intestinal worms, malaria, scabies, boils, and pellagra. When Taichu was flooded in 1944 its patients went to Inrin and then Toroku. He describes both doctors as giving ‘lectures’, Allinson telling ‘yarns’ about his time in India and Glover talking on medical matters. Allinson is often a comic figure in Clement’s eyes, a keen surgeon and ‘the galloping major’ who played the accordion. Glover is the ‘junior’, a good doctor who teaches the orderlies, talks to patients, gives Clement an egg to eat, and sets him to work cleaning ‘a gross of rusty surgeon’s needles’. Glover is ‘a ginger-haired lad…a gentleman… He worked hard – gave away his Red Cross stuff to bad cases and won over even the Nips.’ He discusses his diagnosis of tuberculosis with Glover and sketches his portrait on his 29th birthday (Figure 8).

In November 1944, Clement met survivors of the *Hokusen Maru*, including doctors. In January 1945, his tuberculosis meant he was transferred by bullock cart, train, and lorry to Shirakawa hospital, where he remained until liberation, treated by American Captain Donovan, and also naming Kern, Heinbach, Poweleit, and Waters, with Glattly their senior doctor. He mentions the arrivals of Lewis (‘the Welsh MO’), Seed and Wheeler and wishes Wheeler was treating him, but does not want to antagonise his American doctors.

## Consequences of captivity

Two of the forty-five doctors died in captivity. As mentioned above, William Kennedy died in Heito of dysentery in September 1942, soon after arriving in Taiwan. Australian Colonel Douglas Pigdon died only weeks before the end of the war in Mukden, Manchuria, one of the senior officers moved north when the American bombing campaign intensified. Other doctors were seriously ill during their captivity. Blair was admitted to his own hospital in Taihoku shortly after arriving in Taiwan with ‘a bad go of beri-beri’ which lasted several weeks.^127^ Long after the war, in 1977, he was awarded a war disability pension on account of ‘duodenal ulcer, bacillary dysentery, beri beri, and helminthiasis’.^128^ Wheeler and Seed both became ill with beri beri and dysentery at Kinkaseki and Seed believed his life was saved by the care given by Wheeler and the medical orderlies, which included eight small blood transfusions in March 1945.

Forty-three doctors survived and Appendix 1 shows that, although the two US Navy doctors died soon after the war, others lived to a great age. Some obituaries include a cryptic mention of the long-term effects of captivity but it is not possible to quantify this for such a small group. Robson *et al.* have summarised the epidemiological evidence for an early post-war excess of mortality from tuberculosis, liver disease and accidents, and prolonged morbidity from a variety of causes, including *Strongyloides* infection, neurological complications of malnutrition, and symptoms that would now be termed post-traumatic stress disorder.^129^ Jones and Wessely have reviewed the evolving conceptualisations of the complex psychological consequences of captivity.^130^ These outcomes were more pronounced than seen in former PoWs in the European theatres or the Korean War.

Prisoners of the Japanese were far more isolated than Britons and Americans in German camps and this compounded the mental effects of the extreme physical deprivation and ill-health they experienced for three and a half years. The isolation was not only by race and culture. The Japanese allowed only limited correspondence and at times deliberately withheld mail.^131^ Piper’s first letter arrived in March 1944 and Clement’s in May 1944, posted in November 1942.^132^ Blair recorded only three distributions of Red Cross parcels at Taihoku, in April 1943, May 1944 and May 1945.

Despite their privations, all the surviving doctors in the group described here seem to have returned to their peacetime professional lives after the war. One can speculate that the fact that they continued to practise their profession in the camps might have mitigated the psychological effects of captivity.^133^ Several accounts imply that there were a few paradoxical, almost spiritual, benefits of being a PoW doctor. Seed reflected after the war that it gave him insights into the value of the monastic life. He told Wheeler’s daughter, Anne, that, ‘it was a very character forming experience… My army experience was more valuable to me than my university. Far more valuable. I received an education as a Japanese prisoner of war, quite different from my other experience. And that’s my considered opinion.’

## Footnotes

1

David Piper , I am Well, Who are You? (Exeter: Anne Piper, 1998), 22.

2 David Tett, *Volume 5: The Philippines and Taiwan 1942-1945*, in *A Postal History of the Prisoners of War and Civilian Internees in East Asia during the Second World War* (Wheathampstead: BFA Publishing, 2002–10), xv.

3 Geoffrey Gill, ’[Review] Charles G Roland , Long Night’s Journey into Day (Waterloo, Ontario: Wilfred Laurier University Press, 2001 .’ *Medical History*, 46 (2002), 603–4.

4

Sibylla Jane Flower , ‘Captors and Captives on the Burma-Thailand Railway’, in Bob Moore and Kent Fedorowich (eds), Prisoners of War and their Captors in World War II (Oxford: Berg, 1996).

5

Mary Ellen Condon-Rall and Albert E Cowdrey , ‘Medical Service in the War against Japan’, in The Medical Department (Washington DC: Center of Military History, United States Army, 1998), 363–83.

6 Sibylla Jane Flower, ‘Memory and the Prisoner of War Experience’, in Karl Hack and Kevin Blackburn (eds), *Forgotten Captives in Japanese-Occupied Asia* (London: Routledge, 2008), 62, 64; M.M. Baird, *Allied Prisoners of War Captured in the Far East and Hong Kong* [Incomplete draft of unpublished narrative], The National Archives (TNA): CAB/101/199.

7 ‘Coping with Crisis, Medicine and Disease on the Burma Railway 1942-1945*’* (unpublished PhD thesis: University of Liverpool, 2009); Geoffrey V. Gill and Meg Parkes , Burma Railway Medicine (Lancaster: Palatine, 2017).

8

Rosalind Hearder , Keep the Men Alive (London: Routledge, 2020).

9

Sarah Kovner , Prisoners of the Empire (Cambridge MA: Harvard University Press, 2020).

10

Gregory Michno , Death on the Hellships (Barnsley: Leo Cooper, 2001).

11 Tett, *op. cit.* (note 2).

12

Suzannah Linton (ed), Hong Kong’s War Crimes Trials (Oxford: Oxford University Press, 2013).

13

Seiji Shirane , Imperial Gateway (Ithaca: Cornell University Press, 2022).

14 Michno, *op. cit.* (note 10), 1.

15 Ikuhiko Hata, ‘From Consideration to Contempt’, in Moore and Fedorowich, *op. cit.* (note 4).

16 Flower, *op. cit.* (note 4), 235–40.

17 Translations of official Japanese documents held in the US National Archives and Records Administration (US NARA) quoted by Michael D Hurst, *Never Forgotten* (Taipei: Mei-Hua Publishing, 2020), 103–4, no citation.

18

Julien M. Goodman , M.D.P.O.W. (New York: Exposition, 1972), 130.

19 Piper, *op. cit.* (note 1), 41.

20

W.S. Kent Hughes , Slaves of the Samurai (Melbourne: Oxford University Press, 1947), 138.

21

Frederick Stallard , Mug Shots (Glossop: Paul Bush, 1998).

22
*War Crimes in the Far East. Defendants Yoshimi Taneoshi et al* and *Defendants Uete Taichi et al*, TNA: WO/235/1099 and 235/1105; Linton, *op. cit.* (note 12).

23 Michno, *op. cit.* (note 10), 309-17.

24
*Ibid*, 309–17; Michael Sturma , ‘Japanese Treatment of Allied Prisoners During the Second World War’, Journal of Contemporary History, 55, 3 (2020), 514–34.

25 Hurst, *op. cit.* (note 17).

26 Linton, *op. cit.* (note 12), 86; Hurst, *op. cit.* (note 17), 56. In this field, numbers (eg of PoWs or deaths) are often contested and vary according to the source.

27
powtaiwan.org; *ibid.*

28 Tett, *op. cit.* (note 2), 161–5.

29
*Ibid*; west-point.org/family/japanese-pow/POWSites-Formosa.

30

Tzen-Fu Yui and Hao-Tsu Chu , ’Possible Factors Affecting the Topography of the Backbone Range of Taiwan’, Western Pacific Earth Sciences, 2, 2 (2002), 105–18.

31
powtaiwan.org; *Prisoners of War, Far East: Taiwan POW Camp; Name List, as of 1 November 1944*, TNA: WO/361/1968; character translated by Takehiko Kariya, University of Oxford.

32 Where a date or biographical detail is given without a footnote, the information came from *Ancestry*, *Findmypast*, the *Medical Directory*, or a published obituary, but the citation is omitted for brevity.

33
*Japanse Interneringskaarten KNIL en Marine 1942-1945*, Dutch National Archives.

34

Donald McDonald , Surgeons Twoe and a Barber (London: Heinemann, 1950).

35 Anonymous, *Allinson, Francis Ward*, in Royal College of Surgeons, *Plarr’s Lives of the Fellows*: livesonline.rcseng.ac.uk.

36 Anonymous, *The Sinking of the Centaur*, Australian War Memorial: awm.gov.au/articles.

37
*Information on sinking of SS Tanjong Penang and SS Kuala carrying evacuees from Singapore*, TNA: CO/980/237; *Malaya, Borneo and Sarawak: deaths from enemy action … and on ships*, TNA: RG/33/132.

38 John Keegan, *The Face of Battle* (London: Cape, 1976), 27–36.

39

Frances Houghton , The Veterans’ Tale (Cambridge: Cambridge University Press, 2019).

40 ‘Medical Conditions among Prisoners of War in the Far East’ (unpublished MD thesis: University of Cambridge, 1949), 1.

41 Michael D. Hurst, personal communication.

42
*Dr Benjamin Wheeler Personal Papers 1936-1950*, University of Alberta Archive: UAA-1977-001. Files are closed.

43 Anne Wheeler, *A War Story* (National Film Board of Canada, 1981): nfb.ca/film/war_story

44
*Private Papers of Captain P G Seed*, Imperial War Museum: IWM:Documents.1615. Subsequent Seed quotations are from this note but are not cited for brevity.

45 For example, Robert Collins, ‘A Man Sent from God’, *Reader’s Digest* (August 1983), 11–12, 143–7; Jack Edwards, *Banzai! You Bastards!* (London: Souvenir Press, 1990).

46 Katherine M. Venables, ‘Captain George Blair RAMC’, *Journal of Medical Biography*, 0, 0 (2024). doi:10.1177/09677720241266313

47
*Lieut. George Blair*, Museum of Military Medicine: RAMC/PE11/890/BLAI; *Capt. George Blair, RAMC*, Wellcome Collection: PP/GBL; *Medical records of prisoners of war … kept by Captain George Blair, RAMC*, Wellcome Collection: RAMC/1280; *George Blair*, RAMC Headquarters (HQ) Collection (no accession number).

48
*Papers of John Francis Clement*, Glasgow City Archives: TD/1764. Subsequent Clement quotations are from this note but are not cited for brevity.

49
*What Knots*, 2, 11 (17 September 1945). Blair family papers, copies in the author’s collection.

50 TNA: WO/235/1099 and 235/1105, *op. cit.* (note 22); Linton, *op. cit.* (note 12); Suzannah Linton. *Hong Kong’s War Crimes Trials.* HKU Libraries Digital Initiatives. hkwctc.lib.hku.hk

51 Wellcome: RAMC/1280 and RAMC HQ, *op. cit.* (note 47); Glover, *op. cit.* (note 40).

52 John Bennet, ‘Introduction’, in Dean A Smith and Michael F A Woodruff, *Deficiency Diseases in Japanese Prison Camps* (London: HMSO, 1951), 1–9.

53 Wibb E Cooper, *Medical Department Activities in the Philippines from 1941 to 6 May 1942, and Including Medical Activities in Japanese Prisoner of War Camps* (Washington DC: Office of the Surgeon General, 1946).

54 William Hallock Braddock, *I Cannot Rest from Travel* (New York: Vantage, 1961); Goodman, *op. cit.* (note 18); Robert C Gaskill , Guests of the Son of Heaven (New York: Vantage, 1976); Alvin C Poweleit , USAFFE (Newport KY: A C Poweleit, 1975); Herbert W Coone , Barney M Barco and Lucille Barco Coone , The Sequential Soldier (Baltimore: Gateway, 1992); William Nelson Donovan and Josephine Donovan, *P.O.W. in the Pacific* (Wilmington DE: SR Books, 1998).

55 For example, Edwards, *op. cit.* (note 45); Diary of Lionel Haylor. In Norman Cliff (ed), *Captive in Formosa* (Rochford, Essex: Rochford Press, 1993).

56

William C. Braly , The Hard Way Home (Washington: Infantry Journal Press, 1947); D Clayton James (ed.), *South to Bataan, North to Mukden (Baton Rouge* : University of Georgia Press, 1971); Kent Hughes, *op. cit.* (note 20); Piper, *op. cit.* (note 1); Stallard, *op. cit.* (note 21).

57
*Drawings from the camps in the occupied Dutch East Indies (1942-1945).* The Memory. geheugen.delpher.nl/en/geheugen/pages/collectie/Kamptekeningen.

58 Piper, *op. cit.* (note 1), 39.

59 USS *Block Island* Association, *Formosa POW Rescue.* ussblockisland.us/memories

60 James Morris, *Drawings*, Imperial War Museum: Art.IWM/ART/LD/5527, 5534–9.

61
*Prisoners of war, Far East: Royal Army Medical Corps, Army Dental Corps, Indian Medical Service personnel; pilot roll*, TNA: WO/361/2180.

62
*Prisoners of war, Far East: ’number’ parties transferred overseas and overland (OVS and OVL), 1942; from Singapore*, TNA: WO/361/2068.

63 Wellcome: RAMC/1280, *op. cit.* (note 47).

64 Piper, *op. cit.* (note 1), 54.

65 George Patterson. *A Spoonful of Rice with Salt.* Durham: Pentland Press, 1993, 55.

66 Hurst, *op. cit.* (note 17), 131–2.

67

M.F. Snape , The Royal Army Chaplains’ Department 1796-1953 *(* Woodbridge: Boydell Press, 2008).

68 Wellcome: PP/GBL. *op. cit.* (note 47).

69 Bennet, *op. cit.* (note 52); Gill and Parkes, *op. cit.* (note 7).

70 Kent Hughes, *op. cit.* (note 20), 144.

71 Wellcome: RAMC/1280, *op. cit.* (note 47); Cliff, *op. cit.* (note 55), 38.

72 RAMC HQ, *op. cit.* (note 47).

73
*Ibid.*

74 Patterson, *op. cit.* (note 65), 79.

75 RAMC HQ, *op. cit.* (note 47).

76 Wellcome: RAMC/1280, *op. cit.* (note 47).

77 Gill and Parkes, *op. cit.* (note 7).

78 Piper, *op. cit.* (note 1), 46–7.

79 Glover *op. cit.* (note 40), 11.

80 RAMC HQ, *op. cit.* (note 47).

81 TNA: WO/235/1105, *op. cit.* (note 22).

82 RAMC HQ, *op. cit.* (note 47).

83
*Japan and Taiwan; deaths at various camps.* TNA: WO/361/1475.

84
*Prisoners of war, Far East: List by Major Allinson* and *List by Captain Blair*, TNA: WO/361/1757 and 1758.

85 TNA: WO/361/1475, *op. cit.* (note 83).

86 With the *caveats* about numbers mentioned in note 26.

87 Wellcome: RAMC/1280, *op. cit.* (note 47).

88
*Indian Army Records of Service 1900-1947*, TNA: IOR/L/MIL/14/15396.

89 Wellcome: RAMC/1280, *op. cit.* (note 47).

90
*Ibid.*

91 Wheeler, *op. cit.* (note 42), Anne Wheeler, personal communication; RAMC HQ, *op. cit.* (note 47).

92 Wheeler, *op. cit.* (note 42), Anne Wheeler, personal communication.

93 Michno, *op. cit.* (note 10), 34.

94
*Overseas. Japan Special Party*, TNA: WO/361/2019.

95 Kent Hughes, *op. cit.* (note 20), 117–19.

96

Michael Snape , Forgotten Warrior (London: SPCK, 2023).

97 H.J.D. de Fremery, *Sketches*, Collection Museon-Omniversum The Hague: 116108.

98

Mark Felton , The Coolie Generals (Barnsley: Pen & Sword, 2008); Hamish Ion , ’Brass Hats Behind Bamboo Palisades’, Canadian Journal of History, 46, 2 (2011), 303–31.

99

Raymond Callahan , The Worst Disaster (Newark: University of Delaware Press, 1977).

100 Braddock, *op. cit.* (note 54), 146.

101
*Raggle Taggle: A Collection of Stories, Poems, Articles, Sketches, and Drawings: Compiled from the Magazine Produced under Unusually Difficult Conditions and with Inferior and Unsuitable Materials in the Prisoner of War Camp at Shirakawa, Formosa, during the Year 1944* (Aldershot, Hampshire: Gale & Polden, 1947).

102
*Ibid.*, 102.

103 A.E. Percival, ‘Operations of Malaya Command, from 8^th^ December 1941 to 15^th^ February 1942’, *The London Gazette*, Supplement, 38215 (26 February 1948), 1245–346. Percival’s report was submitted in 1946, and then revised by the War Office.

104 Cooper, *op. cit.* (note 53).

105 Gaskill, *op. cit.* (note 54), 95.

106 Anonymous, ‘Harold W. Glattly, M.D. 1902-1967’, *Journal of Bone and Joint Surgery*, 50 (1968), 431–2; Harold W Glattly , ‘A Statistical Study of 12,000 New Amputees’, Southern Medical Journal, 57, 11 (1964), 1373–8.14195964 10.1097/00007611-196411000-00024

107 James, *op. cit.* (note 56), 65; Jeffrey Grey , ’Alfred Plumley Derham (1891-1962)’, in John Ritchie (ed), Australian Dictionary of Biography (Melbourne: Melbourne University Press, 1993 .

108

Allan Seymour Walker (ed), ‘Volume 2: Middle East and Far East’, in Australia in the War of 1939-1945. Series 5, Medical (Canberra: Australian War Memorial, 1953), 659–63.

109 TNA: WO/361/1757, *op. cit.* (note 84).

110
*Translation of document from the Taiwan PoW camp headquarters, 1 August 1944.* US NARA: RG 238/2015.

111 RAMC HQ, *op. cit.* (note 47).

112 Coone, *op. cit.* (note 54), 162. *Benjo* = toilet.

113 Gaskill, *op. cit.* (note 54), 81.

114 Coone, *op. cit.* (note 54), 162.

115 Goodman, *op. cit.* (note 18), 121.

116 Coone, *op. cit.* (note 54), 163–4; Poweleit, op. cit. (note 54).

117 Goodman, *op, cit.* (note 18), 137.

118
*Ibid.*, 200, 217.

119 Coone, *op. cit.* (note 54), 166.

120 TNA: WO/361/2180, *op. cit.* (note 61).

121 Gill and Parkes, *op. cit.* (note 7); Stanley S Pavillard , Bamboo Doctor (London: Macmillan, 1960).

122
*Report of 5949044 Sgt. Burgess AW*, TNA: WO/361/758.

123 Lewis, in *What Knots*, *op. cit.* (note 49).

124 Piper, *op. cit.* (note 1), 50.

125 Collins, *op. cit.* (note 45); E.E. Leeson, ‘Captain George Blair, RAMC’, *The Gunner*, 104, July (1979).

126 ‘He hath sent me to bind up the broken-hearted, to proclaim liberty to the captives, and the opening of the prison to them that are bound’, Isaiah 61:1; ‘Yea, though I walk through the valley of the shadow of death, I will fear no evil: for thou art with me’, Psalm 23:4.

127 Wheeler, *op. cit.* (note 42), Anne Wheeler, personal communication.

128 Wellcome: PP/GBL, *op. cit.* (note 47).

129

D. Robson , E. Welch , N.J. Beeching and G. Gill , ‘Consequences of Captivity: Health Effects of Far East Imprisonment in World War II’, Quarterly Journal of Medicine, 192 (2009), 87–96 10.1093/qjmed/hcn13718854350.

130

Edgar Jones and Simon Wessely , ‘British Prisoners-of-War: from Resilience to Psychological Vulnerability: Reality or Perception’, 20th Century British History, 21, 2 (2010), 163–8320695409 10.1093/tcbh/hwp056.

131 Tett, *op. cit.* (note 2).

132 Piper, *op. cit.* (note 1), 39.

133 Edgar Jones, personal communication.

## Appendix A: The Taiwan doctors

### Australian Army Medical Corps

Colonel Derham, Alfred Plumley (1891–1962)*

Colonel Pigdon, Douglas Clelland (1891–1945)*

Colonel White, Edward Rowden (1881–1963)*

Captain O’Donnell, Patrick Neil (1916–73)*

### Royal Netherlands Indies Army Medical Service

Maj-General van Rees, Jacob (1888–1962)

Colonel Goudswaard, Adriaan (1892–?)

Colonel Spier, Marcus (1890–?)

Colonel van Manen, Sipke Evert (1889–1965)

Lieutenant Versnel, Frits (1914–2003)

### Royal Army Medical Corps

Brigadier Stringer, Charles Herbert (1886–1961)*

Colonel Bennet, John (1893–1976)*

Colonel Simson, John Thomas (1884–1976)

Colonel Wardle, Valentine Hutchinson (1886–1968)*

Captain Blair, George (1916–79)^†^

Captain Glover, John Arnold (1915–97)

Captain Grant, William Russell (‘Russ’) (1910–87)*

Captain Lewis, Cyril Vernon (‘Lew’) (1912–2001)^‡^

Captain Seed, Peter Gerard (1913–77)^†^

### Indian Medical Service

Colonel Mitchell, John Marsters (1889–1975)*

Lt-Colonel Kennedy, William Graeme (1909–42)*

Major Allinson, Francis Ward (1904–90)

Major Wheeler, Benjamin Morrill (‘Ben’) (1910–63)^†^

### Royal Naval Medical Service

Surgeon-Commander Tincker, Robert Wise Holden (1899–1981)

### US Army Medical Corps

Colonel Braddock, William Hallock (1887–1968) *Lima Maru* September 1942

Colonel Cooper, Wibb Earl (1886–1983) *Nagara Maru* August 1942

Colonel Gillespie, James O (1902–92) *Lima Maru* September 1942

Lt-Colonel Gaskill, Robert C (1902–76)^‡^

Lt-Colonel Glattly, Harold Willard (1902–67) *Nagara Maru* August 1942

Major Raulston, John William (1907–98)^‡^

Major Ruth, Weldon Kenneth (1912–62)^‡^

Captain Coone, Herbert William (‘Herb’) (1912–2001)^‡^

Captain Donovan, William Nelson (1910–98)^‡^

Captain Goodman, Julien Max (1913–2006)^‡^

Captain Heinbach, Wilfred Francis (1906–91)^‡^

Captain Kern, Clyde Vincent (‘Ted’) (1911–62)^‡^

Captain Merkel, Emil Elvin (1907–2001)^‡^

Captain Poweleit, Alvin Charles (1908–97)^‡^

Captain Schneider, Leo Victor (1907–97)^‡^

Captain Vande Velde, Joseph Daniel (1909–75)^‡^

Captain Waters, William Isaac (1912–50)^‡^

Captain Wilson, William E (?–?)^‡^

Lieutenant Chamberlain, George Earle (1913–99)^‡^

Lieutenant Rose, William D (1913–89)^‡^

### US Navy Medical Corps

Captain Davis, Robert Gaylord (1883–1948)

Captain Lowman, Kenneth Earle (1890–1946)

* In ‘Special Party’ from Singapore on *England Maru* and *Fukkai Maru* (O’Donnell) August 1942

^†^ In Overseas Party ‘Z’ from Singapore on *England Maru* October 1942

^‡^ In a group from Manila on *Hokusen Maru* October 1944

